# Supplementary material for: Monogenic disorders of immunity: Common variants are not so rare
Source: Cell Genom. 2026 Jan 8;6(1):101129. doi: 10.1016/j.xgen.2025.101129 (PMC12926205; doi:10.1016/j.xgen.2025.101129)
Supplement: Document S2. Article plus supplemental information [file mmc2.pdf]

Cell Genomics

CellPress
OPEN ACCESS

Perspective

Monogenic disorders of immunity: Common variants are not so rare

Vivien Béziat[1,2,3,*] and Jean-Laurent Casanova[1,2,3,4,5,*]
[1]Laboratory of Human Genetics of Infectious Diseases, Necker Branch, INSERM, Necker Hospital for Sick Children, Paris, France
[2]Imagine Institute, Paris-Cité University, Paris, France
[3]St. Giles Laboratory of Human Genetics of Infectious Diseases, Rockefeller Branch, The Rockefeller University, New York, NY, USA
[4]Pediatric Hematology-Immunology and Rheumatology Unit, Necker Hospital for Sick Children, AP-HP, Paris, France
[5]Howard Hughes Medical Institute, The Rockefeller University, New York, NY, USA
*Correspondence: vivien.beziat@inserm.fr (V.B.), casanova@rockefeller.edu (J.-L.C.)
https://doi.org/10.1016/j.xgen.2025.101129

SUMMARY

According to the current paradigm, human monogenic disorders underlying immunological phenotypes are due to rare (frequency <1%) as opposed to common (>1%) alleles. However, as reviewed here, an increasing number of studies have reported monogenic disorders of immunity, recessive or dominant, involving alleles that are currently common in specific small or large populations. Examples range from *IFNAR1* and *IFNAR2* null alleles in the Arctic and Pacific to *PTCRA* hypomorphic alleles in South Asia. This situation may be explained by a history of (1) population bottlenecks followed by expansion; (2) genetic drift before the advent of an environmental trigger; (3) slow purging, especially for recessive, mild, or incompletely penetrant conditions; and/or (4) balancing selection with a heterozygous advantage. In patients with suspected monogenic immunological conditions, a role for alleles common in the corresponding population should not be excluded. At odds with the prevailing view, common alleles may underlie monogenic disorders of immunity and should therefore be considered.

INTRODUCTION

Human inborn errors of immunity (IEIs) were originally described, during the period from 1946 to 1952,[1–3] as Mendelian traits—monogenic disorders with complete penetrance. If not all carriers of an at-risk genotype display the corresponding phenotype, then penetrance is incomplete, and monogenic inheritance can be referred to as "non-Mendelian." Since the late 1990s, the description of incomplete penetrance for disease-causing immunological phenotypes, and even more frequently for the clinical phenotypes themselves, has progressively blurred the initially described Mendelian nature of these conditions. A number of "non-Mendelian monogenic disorders" have been described for infection, autoinflammation, autoimmunity, allergy, and cancers.[4] When at least two kindreds are available, the relative risk (RR), or odds ratio (OR) of developing a phenotype of interest can be estimated and should be higher than an arbitrary but conservative threshold in carriers of the at-risk genotype. The concepts of RR and OR, as well as the relationship between RR and penetrance, are explained in Box 1. Gaining an understanding of the mechanisms underlying incomplete penetrance of monogenic disorders is a major endeavor in the field of IEI and human genetics at large. A handful of studies have yielded promising results in this respect[5–7] and are detailed in Box 2.

Here, we postulate that a disease can be considered monogenic even with low penetrance, provided that it is driven by a monogenic genotype, as supported by genetic data (i.e., association of genotype and phenotype in a multiplex kindred or, better, in multiple kindreds) or experimental (i.e., molecular and cellular mechanism connecting genotype and phenotype, which is required for single-patient studies and preferable for single-kindred studies).[19,20] This definition does not exclude the possibility that an allele conferring high risk with low penetrance may require as yet unknown alleles at a modifier locus to underlie the phenotype, as explained in Box 2. Admittedly, the distinction between monogenic and digenic can become arbitrary; the respective contributions of the alleles at the two loci (e.g., their frequency, functional impact, or both) may tip the balance in favor of either term. Moreover, there is no universally accepted "monogenic threshold," but an OR/RR $\geq$ 5 seems to be a reasonably conservative threshold in this context, both biologically and clinically, and is used in this review.

Most monogenic disorders of immunity were discovered via patient- and family-based studies or studies of rare conditions in the field of IEI. However, a subset of monogenic immunological conditions was discovered in large population-based studies.[21–25] In these studies, focusing on common conditions, the involvement of a common allele was expected. By contrast, regardless of their penetrance, patient-based IEIs have been widely considered, since the discovery of the first genotypes in 1985,[26,27] as being due to rare alleles, with a minor-allele frequency (MAF) of <0.01 across the populations studied. This notion is consistent with the rarity of the original IEI-defining traits themselves. Consequently, most exome or

This is an open access article under the CC BY license (http://creativecommons.org/licenses/by/4.0/).

Box 1. Effect size, RR, OR, and penetrance

The effect size quantifies how much a genetic variation influences a particular characteristic, such as disease risk. RR and OR are classical measures of effect size of a variant for a binary trait (e.g., affected/unaffected). The RR between groups is calculated as the ratio of the penetrance of the phenotype of interest between the two groups. The penetrance in a group (e.g., carriers or non-carriers of an at-risk genotype) is the observed frequency of the phenotype in the group and is denoted here as f. If group A (carriers) has a penetrance of f_a and group B (non-carriers) a penetrance of f_b, then the RR of the phenotype in group A relative to group B is f_a/f_b. If, for example, $f_a = 0.2$ and $f_b = 0.01$, then RR = 20. In some circumstances, there is a linear relationship between the RR and f_a.[8] By contrast, the OR between groups is calculated as the ratio of the odds of the two groups. The odds of group A are $f_a/(1-f_a)$. The odds of group B are $f_b/(1-f_b)$. Thus, the OR of group A relative to group B is $(f_a/(1-f_a))/(f_b/(1-f_b))$. In our example, the OR = (0.2/0.8)/(0.01/0.99) = 24.75. The RR should be preferred over the OR where possible, but, unlike the OR, it is often impossible to calculate in case-control studies. For rare events (e.g., diseases), the OR usually provides a good approximation of the RR. An OR or RR close to 1 indicates a lack of association between the genotype and phenotype considered. A low or high OR or RR, deviating significantly from 1, is suggestive of a negative or positive association, respectively. Nevertheless, neither implies causality or provides any information about the underlying mechanism. Causality can be inferred from existing biological or medical knowledge or from additional, mechanistic experiments at the molecular, cellular, or whole-organism level.

genome pipelines in both research and diagnostic laboratories performing analyses on individual patients and families currently filter out common alleles, defined as those with a MAF >0.01. Here, we adopted a MAF of 0.01 as the cutoff between rare and common alleles, even though such a threshold is arbitrary, because this is the most widely used threshold and, as such, the most appropriate for our purpose of revisiting its usefulness. These filters consider the global MAF, or the highest MAF, or the MAF in the corresponding population—typically one of the seven major ancestries, more rarely a smaller population.

Of course, there is also no “size threshold” for defining a population, and one could even provocatively assert that any disease-causing allele is common in any affected kindred, treated as an ultra-small population. More reasonably, a hamlet or a village could justifiably be considered to contain a population, particularly if geographically isolated—on a small island or in a mountain valley, for example. We review here the known common alleles underlying monogenic immunological conditions, including both IEIs identified in patient-based studies and conditions identified in studies of populations (Figure 1). The high frequency of theses alleles may be theoretically explained by a history of (1) population bottlenecks followed by expansion; (2) genetic drift before the advent of an environmental trigger; (3) slow purging, especially for recessive, mild, or incompletely penetrant conditions; and/or (4) balancing selection with a heterozygous advantage. In most cases, their high frequency remains unexplained. We divide these variants into two groups, based on high and low penetrance, in which they are classified chronologically.

Box 2. Possible causes of incomplete penetrance for monogenic disorders

Documented and suggested mechanisms of incomplete penetrance include (1) environmental factors, as a lack of or insufficient exposure to environmental triggers, including pathogens, allergens, and carcinogens, can obviously account for a lack of phenotype in at-risk individuals (e.g., in IL-12Rβ1-deficient individuals, BCG disease cannot occur in the absence of BCG vaccination[9]); (2) broad, pre-existing adaptive immunity to the same or a related pathogen, as the recognition of the invading microbe by T or B cells can mitigate a genetic deficiency of innate or intrinsic immunity (e.g., BCG disease protects IL-12Rβ1-deficient individuals against environmental mycobacterial disease[9]); (3) narrow, pre-existing humoral responses to a specific microbial virulence factor can even mask the innate genetic disorder (e.g., antibodies against lipoteichoic acid [LTA] prevent staphylococcal disease in TIR Domain Containing Adaptor Protein [TIRAP]-deficient individuals, whose defect renders them susceptible exclusively to LTA-expressing staphylococci[6,7]); (4) non-random monoallelic expression, which can lead to expression of the wild-type and mutant alleles in healthy and sick heterozygotes, respectively (e.g., selective expression of the mutant *Phospholipase C Gamma 2* [*PLCG2*] allele in the B cells of affected heterozygous carriers, leading to antibody deficiency[10]); (5) somatic mosaicism, as the reversion of the germline defect in relevant cell lineages can improve the condition (e.g., reversion to wild-type of one mutant allele in the B cells of a patient with ADA deficiency progressively improves the clinical phenotype[11]); (6) modifier genes, as germline epistasis can govern the penetrance of single-gene lesions (e.g., a common allele of *Bone Morphogenetic Protein 2* [*BMP2*] greatly increases the risk of developing craniosynostosis in patients with deleterious *SMAD Family Member 6* [*SMAD6*] alleles[12–15]); and (7) and regulatory variants in *cis* of a heterozygous variant that influence the expression level of the mutant or wild-type allele.[16–18]

PATHOGENIC ALLELES WITH HIGH PENETRANCE

Common variants of *C2* can underlie infection or autoimmunity

Patient-based studies led to the discovery of common genetic defects of complement.[28] There are three complement activation pathways: the classical, alternative, and lectin pathways.[29] The classical pathway is activated by the Fc fragment of an immunoglobulin (IgG1, IgG2, IgG3, or IgM) linked to an antigen. The lectin pathway, homologous to the classical pathway, is activated by MBL (mannose-binding lectin) or ficolins, which recognize mannose residues on the surface of pathogens. The alternative pathway is directly activated by binding of the C3 protein to pathogens. Each pathway has its own cascade of consequences, but all three converge to activate the terminal pathway, leading to formation of the membrane attack complex (MAC). MAC assembly results in the formation of pores in the lipid membranes of pathogens, particularly Gram-negative bacteria, leading to their lysis. Genetic deficiencies of MAC components underlie invasive disease due to *Neisseria*.[28,29] Complete deficiencies of alternative pathway proteins confer a predisposition to invasive bacterial infections.[28,29] Deficiencies of the lectin pathway are not associated with a well-defined clinical phenotype.[30] Deficiencies of the classical pathway underlie invasive bacterial infections and systemic lupus erythematosus.[31,32]

Deficiency of C2, a crucial molecule in the lectin and classical complement pathways, was first described in 1966.[33] It was later

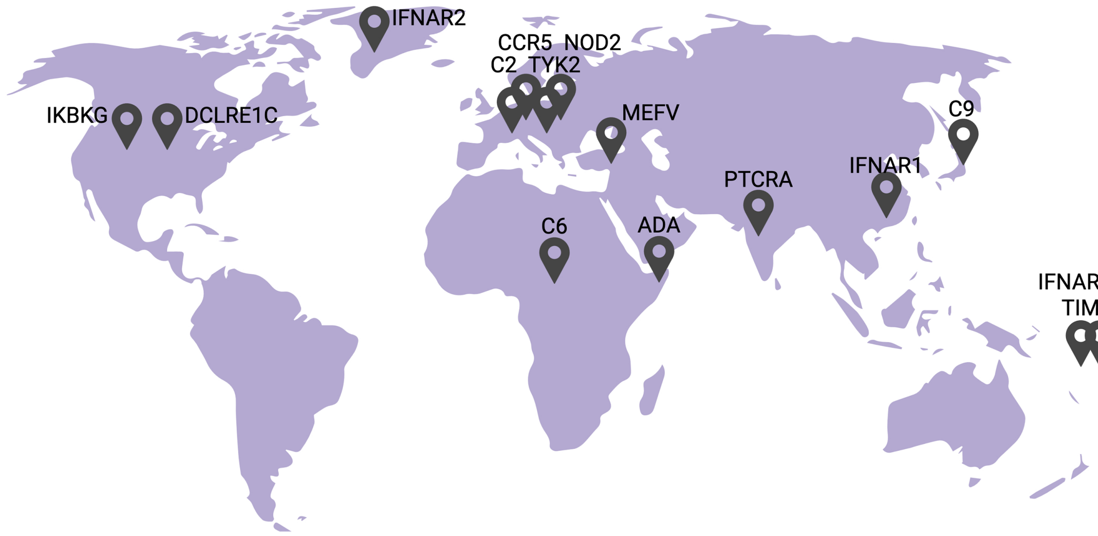

Figure 1. Worldwide distribution of frequent variants underlying IEIs

Complete C2 deficiency underlies invasive bacterial infection and systemic lupus erythematosus and is frequent in Europeans. Complete C6 and C9 deficiencies underlie invasive *Neisseria* disease and are frequent in sub-Saharan Africans and Japanese, respectively. MEFV GoF variants underlie FMF and are particularly prevalent in Arabs, Armenians, Jews, and Turks. Complete NOD2 deficiency underlies Crohn's disease with low penetrance and is frequent in Europeans. Complete deficiencies of ADA, DCLRE1C, and IKBKG underlying SCID are frequent in Somalians, Native Americans (Apache and Navajo) and Northern Cree Canadians, respectively. CCR5 deficiency is frequent in Europeans, where it protects against HIV-1 infection but confers a predisposition to WNV disease. Partial TYK2 deficiency underlies TB and is frequent in Europeans. Complete TIM3 deficiency underlies SPTCL and HLH with low penetrance and is frequent in East Asians and Polynesians. Complete deficiencies of IFNAR1 and IFNAR2 underlie severe viral infections and are frequent in Western Polynesians and Arctic populations, respectively. Partial IFNAR1 deficiency underlies viral infections and is frequent in South Han Chinese. Partial PTCRA deficiency underlies autoimmune phenotypes and is frequent in South Asia and the Middle East.

recognized as the most common complement protein deficiency in the European population.[34] In 90% of cases, it is caused by a deletion of 28 bp (rs9332736) in *C2*, leading to skipping of exon 6 and a complete absence of C2 protein synthesis.[35] The allele frequency for this deletion is ~0.007 in Europeans and up to 0.01 in Ashkenazi Jews, according to gnomAD v.4.1[36] (Table 1). The clinical penetrance of C2 deficiency is incomplete and may be overestimated due to observation bias.[37] In a Swedish series of 40 C2-deficient patients, 34 patients were homozygous for the 28 bp deletion (rs9332736) in *C2*, and three others were compound heterozygous for this and another deleterious variant. About 25% of the patients had systemic lupus erythematosus or a related autoimmune disease, and about 60% of them had a history of invasive infection. For the individuals with invasive infection, 64.3% of the cases of meningitis and 52.2% of the cases of septicemia were due to *Streptococcus pneumoniae*.[38] Three patients in this series (7.5%) had a history of meningococcal disease—meningitis in two cases and sepsis in the third. Over a mean observation time of 39 years, six of the 40 patients (15%) developed only minor infections and had no autoimmune phenotype at their last follow-up visit, suggesting a clinical penetrance of at least 85% in a lifetime (Table 2).

Common variants of *C6* or *C9* can underlie infection

MAC proteins include the C5, C6, C7, C8α, C8β, C8γ, and C9 proteins.[81] Inherited deficiencies of all MAC proteins except C8γ have been reported in humans. Hundreds of patients with MAC deficiency have been reported since the description of the first case in 1976.[82–84] Individuals with deficiencies of the C5-C8 MAC proteins present a narrow spectrum of susceptibility to infection, limited to bacteria of the genus *Neisseria* (*Neisseria meningitidis* and *Neisseria gonorrhoeae*)[41] and, more rarely, *Haemophilus parainfluenzae*.[85,86] These genotypes result in a complete inability to form the MAC and an increase in the risk of infection by a factor of 7,000–10,000 with at least one episode of meningococcal disease in 50%–60% of at-risk genotype carriers (Table 2).[41,69] C6 deficiency was first described in 1974 in a single kindred,[87] and a high prevalence of C6 deficiency in African-Americans was reported in 1984.[41] Three variants of *C6* account for most of the MAC protein deficiencies reported in Africans: p.Gln274Argfs*46 (rs557023458), p.Gln380Serfs*7 (rs375762365), and p.Asp627Thrfs*4 (rs61469168).[42,88,89] The allele frequencies of these variants in Africans and African Americans in gnomAD v.4.1 are 0.0042, 0.0068, and 0.0108, respectively (Table 1). Based on the cumulative frequencies of the three major alleles, 1 in 2,500 Africans has a complete deficiency of C6. As a result, the African population has a particularly strong predisposition to meningococcal meningitis. Indeed, Africa, with its sadly famous "meningitis belt," is well known to be the region of the world with the highest prevalence of meningitis and an incidence of up to 1 in 1,000.[90,91]

C9 deficiency was first described in 1979 in a single Japanese kindred.[92] C9 deficiency confers a predisposition to invasive *Neisseria* disease but to a lesser extent than other terminal pathway deficiencies affecting the C5–C8 proteins.[43,44] Indeed, during formation of the MAC, C9 allows enlargement of the pore but is not

Table 1. Common alleles underlying monogenic predispositions to infection, autoimmunity, or autoinflammation

Gene (product)[a]	SNP	Chr.	Position (GRCh38)	Reference	Alt.	Consequence (Mane selected transcript)	Heritability	Populations with highest MAF	Highest MAF	Clinical phenotype	Reference
ADA	rs1057520217	20	44651601	G	A	p. Gln3*	recessive	Somalians	0.024	SCID	Sanchez et al.,[39] Adams et al.[40]
C2	rs9332736	6	31934288	ATGGTG GACAGG GTCAGG AATCAG GAGTC	A	skipping of exon 6	recessive	Ashkenazi Jewish (gnomAD v.4.1)	0.01135	*Neisseria* meningitis (high penetrance)	Johnson et al.[35]
C6	rs61469168	5	41158762	TC	T	p.Asp627Thrfs*4	recessive	African (gnomAD v.4.1)	0.01080	*Neisseria* meningitis (high penetrance)	Ross et al.,[41] Nishizaka et al.[42]
C9	rs121909592	5	39341276	G	A	p.Arg116*	recessive	Japanese	0.0298	*Neisseria* meningitis (low penetrance)	Nagata et al.,[43] Nishizaki et al.,[44] Kira et al.,[45] Higasa et al.[46]
CCR5	rs333	3	46373452	TACAGT CAGTAT CAATTC TGGAAG AATTTC CAG	T	p.Ser185Ilefs*32	recessive Mendelian	European	0.1315 (gnomAD v.4.1)	WNV (+resistance to HIV-1 infection)	Glass et al.,[24] Lim et al.,[25] Dean et al.,[47] Liu et al.,[48] Samson et al.[49]
DCLRE1C (ARTEMIS)	rs121908157	10	14934461	G	T	p.Tyr199*	recessive	Navajo and Apache Native Americans	0.021	SCID	Murphy et al.,[50] Li et al.[51]
HAVCR2 (TIM-3)	rs184868814	5	157106776	T	C	p.Tyr82Cys	recessive	East Asians (gnomAD v.4.1)	0.01656	HLH and SPTCL	Gayden et al.[52]
IFNAR1	rs72552343	21	33349398	TTCC	T	p.Pro335del	recessive (possibly dominant)	Southern Han Chinese	0.011–0.023	viral infections	Al Qureshah et al.,[53] Zhang et al.[54]
IFNAR1	rs201609461	21	33352770	G	T	p.Glu386*	recessive	Western Polynesians	0.01250	viral infections	Bastard et al.[55]
IFNAR2	rs1987287426	21	33245010	T	C	p.Ser53Pro	recessive	Inuits	0.034	viral infections	Duncan et al.[56]
IKBKB	rs886041036	8	42318603	G	GG	p.Gln432Profs*62	recessive	Northern Cree	0.076	SCID	Pannicke et al.,[57] Rubi et al.[58]
MEFV	rs61752717	16	3243407	T	C	p.Met694Val	recessive (possibly dominant)	Turkish[b] North African Jews	0.017 0,08	FMF	French FMF Consortium,[59] The International FMF Consortium et al.,[60] Shohat et al.,[61] Stoffman et al.,[62] Honda et al.[63]

(*Continued on next page*)

Table 1. *Continued*

Gene (product)[a]	SNP	Chr.	Position (GRCh38)	Reference	Alt.	Consequence (Mane selected transcript)	Heritability	Populations with highest MAF	Highest MAF	Clinical phenotype	Reference
MEFV	rs28940579	16	3243310	A	G	p.Val726Ala	recessive (possibly dominant)	Ashkenazi Jews (gnomAD v.4.1)	0.03914	FMF	Johnson et al.,[35] French FMF Consortium,[59] The International FMF Consortium et al.,[60] Stoffman et al.,[62] Honda et al.[63]
NOD2	rs2066844	16	50712015	C	T	p.Arg675Trp	recessive	non-Finnish Europeans (gnomAD v.4.1)	0.04766	Crohn's disease	Ogura et al.,[21] Hugot et al.,[22] Hampe et al.,[23] Ahmad et al.,[64] Bonen et al.[65]
NOD2	rs2066845	16	50722629	G	C	p.Gly881Arg	recessive	Middle East (gnomAD v.4.1)	0.03728	Crohn's disease	Ogura et al.,[21] Hugot et al.,[22] Hampe et al.,[23] Ahmad et al.,[64] Bonen et al.[65]
NOD2	rs2066847	16	50729867	G	GC	p.Leu980Profs*2	recessive	non-Finnish Europeans (gnomAD v.4.1)	0.02163	Crohn's disease	Ogura et al.,[21] Hugot et al.,[22] Hampe et al.,[23] Ahmad et al.,[64] Bonen et al.[65]
PTCRA (Pre-TCRα)	rs200942121	6	42923120	A	C	p.Asp51Ala	recessive	South Asians (gnomAD v.4.1)	0.01675	various autoimmune diseases	Materna et al.[66]
TYK2	rs34536443	19	10352442	G	C	p.Pro1104Ala	recessive	non-Finnish Europeans (gnomAD v.4.1)	0.04450	TB	Boisson-Dupuis et al.,[67] Kerner et al.[68]

[a]HLA alleles are not included.
[b]Turkiye National Genome and Bioinformatics Project. Turkish Genome Project Data Sharing Portal v.1.0 (tgd.tuseb.gov.tr/en). Accessed May 23, 2024.

CellPress
OPEN ACCESS

Table 2. Penetrance and RR of IEIs associated with common variants

Gene	Type of deficiency	Transmission model	Penetrance	RR or OR	Reference
ADA	complete	recessive	100%	N.D.	Sanchez et al.,[39] Adams et al.[40]
C2	complete	recessive	>85% (lifetime)	N.D.	Jönsson et al.[38]
C6	complete	recessive	50%–60%	RR = 7,000 to 10,000	Ross et al.,[41] Figueroa et al.[69]
C9	complete	recessive	5%–10% (lifetime, Fukuoka city)	RR ∼ 1,000	Nagata et al.[43]
CCR5	complete	recessive	N.D.	OR = 5	Glass et al.,[24] Lim et al.,[25] Ellwanger et al.[70]
DCLRE1C (ARTEMIS)	complete	recessive	100%	N.D.	Li et al.[71]
HAVCR2 (TIM-3)	complete	recessive	max 0.3%/year[a]	N.D.	Gayden et al.,[52] Kim et al.[72]
IFNAR1	complete	recessive	∼100% (lifetime)	N.D.	Bastard et al.[55]
IFNAR1	hypomorphic	recessive	unknown	N.D.	Al Qureshah et al.[53]
IFNAR1	hypomorphic	dominant	unknown	N.D.	Al Qureshah et al.[53]
IFNAR2	complete	recessive	∼100% (lifetime)	N.D.	Duncan et al.[56]
IKBKB	complete	recessive	100%	N.D.	Pannicke et al.,[57] Cuvelier et al.[73]
MEFV	GoF	recessive	∼100% (for p.Met694Val)	N.D.	French FMF Consortium,[59] The International FMF Consortium et al.,[60] Ben-Chetrit,[74] Gershoni-Baruch et al.[75]
MEFV	GoF	dominant	N.D.	RR = 6–8	Cazeneuve et al.,[76] Medlej-Hashim et al.,[77] Jéru et al.,[78] Eyal et al.[79]
NOD2	hypomorphic	recessive	1.5%	OR = 10–42	Ogura et al.,[21] Hugot et al.,[22] Hampe et al.,[23] Ahmad et al.,[64] Bonen et al.,[65] Yazdanyar et al.[80]
PTCRA (Pre-TCRα)	hypomorphic	recessive	∼12% (Centogene cohort) unknown over lifetime	OR = 5	Materna et al.[66]
TYK2	hypomorphic	recessive	∼80% (lifetime)	OR = 5	Kerner et al.[68]

HLA alleles are not included. N.D., no data.
[a]Calculated based on the incidence of SPTCL in Māori/Pacific people and the allele frequency in Polynesians.[52,72]

required for its formation. In cases of C9 deficiency, complement activity is severely reduced but not abolished. C9 deficiency is frequent in Japan,[93] where the p.Arg116* (rs121909592) loss-of-function variant has a frequency as high as 0.0298[46,94] (Table 1). In Japan, ∼1 person in 1,100 is predicted to have a complete deficiency of C9. Based on data for the city of Fukuoka in Japan, the annual risk of developing meningococcal disease is 1,000 times greater in C9-deficient individuals than in other individuals, with an annual penetrance of 0.1% in C9-deficient individuals versus 0.0001% in other individuals (Table 2).[43] The deleterious alleles of C6 and C9 common in African and Japanese populations may have provided an evolutionary advantage. The MAC is a double-edged sword; it contributes to antibacterial immunity and allows lysis of Gram-negative bacteria, but this lysis is accompanied by release of bacterial components, such as lipopolysaccharide, which can trigger inflammation and septic shock. Würzner hypothesized that partial C6 deficiency would have been an evolutionary advantage in the event of endotoxin shock during infection with Gram-negative bacteria.[95] This hypothesis could be extended to complete C6 deficiency in Africans, C9 deficiency in Japanese, and C2 deficiency in Europeans. Moreover, purging of these alleles may be slow, as they are pathogenic only in the homozygous state. Nevertheless, it is intriguing that MAC protein deficiency confers a predisposition only to *Neisseria* meningitis, suggesting that the MAC is largely redundant against other pathogens, including other Gram-negative bacteria (e.g., *E. coli*, *B. pertussis*, and *V. cholerae*).

Common variants of *MEFV* can underlie autoinflammation

Other family-based studies have focused on familial Mediterranean fever (FMF), the most frequent known genetic autoinflammatory disease worldwide, first described in 1908.[96] It was first shown to be a genetic disease in 1945,[97,98] and biallelic mutations of *MEFV* were reported in 1997.[59,60,99] FMF is caused by homozygosity or compound heterozygosity for gain-of function variants of *MEFV*, encoding pyrin,[59,60,74] with clinical penetrance being incomplete or complete depending on the genotype (Table 2).[75] Heterozygosity for *MEFV* variants may also be a significant risk factor for the development of FMF (RR ∼6–8 times higher than for non-carriers), as about a third of the patients carry only a monoallelic variant.[76–79] In heterozygous

patients, non-random autosomal monoallelic expression of the *MEFV* locus is warranted to be tested, as it might explain incomplete penetrance (Box 2). FMF may therefore be considered a semi-dominant condition with a lower risk and penetrance in heterozygotes. Pyrin promotes assembly of the pyrin inflammasome and interleukin-1 β (IL-1β) secretion.[100] Gain-of function *MEFV* variants impair the binding of the 14-3-3 inhibitory protein to pyrin, promoting uncontrolled inflammasome activation.[101,102] Excessive inflammation in the patients leads to recurrent fever accompanied by serositis (peritonitis, pleuritis, pericarditis, or synovitis). Left untreated, repeated flare-ups of inflammation can lead to secondary amyloidosis, which may cause serum amyloid A (SAA) protein deposition, resulting in kidney failure.[61]

FMF is particularly prevalent in people of Jewish, Armenian, Arabian, or Turkish descent, with the frequency of allele carriage estimated at 20% in these populations.[103–105] Four founding variants of exon 10 account for most cases in populations from the Mediterranean basin: p.Met680Ile (rs28940580), p.Met694Ile (rs28940578), p.Met694Val (rs61752717), and p.Val726Ala (rs28940579). For instance, the p.Val726Ala variant has an allele frequency of 0.039 in Ashkenazi Jews (gnomAD v.4.1) (Table 1). The p.Met694Val allele has a frequency of 0.017 in Turks (Turkish genome project) and 0.08 in North African Jews (Table 1).[62] The p.Met694Val allele is also associated with an increased risk of ankylosing spondylitis in Turks, with an OR of 4.8.[106] It has been suggested that the high prevalence of these alleles in Mediterranean and Jewish populations results from a selective advantage of heterozygosity, which is thought to have provided protection against severe infectious diseases of the past. It was recently suggested that heterozygosity for *MEFV* variants confers cellular resistance to *Yersinia pestis* by decreasing the interaction of MEFV with *Yersinia* outer protein M (YopM), a *Yersinia pestis* virulence factor, but preserving its binding to the WT human pyrin, thereby attenuating YopM-induced IL-1β suppression.[107–109] It is therefore tempting to speculate that heterozygosity for any of these *MEFV* variants may have conferred protection against epidemics of plague, which killed a very large proportion of people in Europe and the Middle East.

Common variants of *ADA*, *DCLRE1C*, and *IKBKB* can underlie severe T cell deficiencies

Patient-based studies of a globally rare immunodeficiency paradoxically revealed causal alleles in small populations in which this deficiency is not that rare. T and B lymphocytes define adaptive immunity and are essential for long-term survival in an ever-changing environment containing a multitude of pathogens. T and B cells differentiate in the thymus and bone marrow, respectively, following a tightly regulated process involving the somatic and clonal rearrangement of the genomic loci corresponding to the T and B cell receptors.[110] Severe combined immunodeficiencies (SCIDs) are a group of Mendelian IEIs defined by a lack of T cell development, which may be associated with deficiencies of other lineages (e.g., natural killer [NK] and B cells).[111] In the absence of hematopoietic stem cell transplantation, SCIDs are invariably lethal due to overwhelming infections during the first year of life.

SCID is very rare in the general population (about 1 in 50,000), but 1 of 2,000 neonates of the Navajo and Apache Native American populations have $T^-B^-NK^+$ SCID. This condition, known as Athabascan SCID (SCIDA) since 1980,[50] was estimated in 1991 to be driven by a single gene variant present in 2.1% of the corresponding population,[112] mapped to chromosome 10 in 1998.[71] The risk of SCIDA is increased by homozygosity for a single-nucleotide substitution in *DNA Cross-Link Repair 1C* (*DCLRE1C*) that was identified in 2002.[51] *DCLRE1C* is a crucial gene for T cell receptor (TCR) and B cell receptor (BCR) rearrangement during the differentiation of T and B cells, respectively.[113] The resulting p.Tyr199* variant (rs121908157) has an allele frequency of 0.021 in the Navajo and Apache populations (Tables 1 and 2). Similarly, $T^-B^-NK^-$ SCID prevalence in Somali neonates is about 1 in 5,000 due to a common premature stop codon in the adenosine deaminase (ADA) gene.[39] ADA is an enzyme that catalyzes irreversible deamination of adenosine and deoxyadenosine to inosine and deoxyinosine and is required to prevent cellular toxicity, in particular in immature lymphocytes. The p.Gln3* variant (rs1057520217) has an allele frequency of 0.024 in Somali people and is hypomorphic. Homozygotes have partial ADA deficiency (Tables 1 and 2).[39,114] Of note, the allele frequency study was performed among Somali migrants in Denmark in 2007. Although an ascertainment bias is unlikely, a larger study in Somalia remains to be performed, as one cannot exclude the possibility that a specific Somali subpopulation emigrated to Denmark.

The nuclear factor κB (NF-κB) pathway plays a major role in signal transduction downstream of many receptors and is involved in many biological processes.[115] The *Inhibitor Of Nuclear Factor Kappa B Kinase Subunit Beta* (*IKBKB*) gene encodes the IKKβ molecule, which plays a major role in the NF-κB canonical pathway. Inherited IKKβ deficiency is associated with a SCID-like infectious phenotype but with normal T cell counts.[57,73] The patients have a combined immunodeficiency (CID). Interestingly, the first patients with IKKβ deficiency to be reported were all of Northern Cree descent and were living in remote communities in the Manitoba and Saskatchewan provinces of Canada.[57] All were homozygous for a frameshift mutation, p.Gln432Profs*62 (rs886041036), which was later shown to have an allele frequency of 0.076 in Northern Cree individuals[58,116] (Table 1). The Apache, Navajo, and Cree populations are relatively small, consisting of ∼300,000, ∼70,000, and ∼200,000 people, respectively. The Somali population is larger, consisting of about 18 million people. DCLRE1C, ADA, and IKBKB deficiencies in these populations neatly illustrate the fact that alleles conferring a predisposition to severe infections can be found at relatively high frequency in specific, small, isolated human populations. The high frequency of these alleles in these populations is unlikely to result from balancing selection. It probably results from a founder effect; in other words, a genetic drift with isolation or bottlenecks followed by rapid expansion of the corresponding populations.[117] Its persistence attests to the slow purging of recessive conditions even when homozygotes die in infancy.

A common *TYK2* variant underlies tuberculosis

Tuberculosis (TB) is an airborne disease typically triggered by *Mycobacterium tuberculosis*. TB is endemic in many countries

and the leading cause of death from a single pathogen.[118] Every year ~10 million people fall ill due to *M. tuberculosis* infection, and 1.3 million individuals die from TB (World health organization, 2022). Mendelian susceptibility to mycobacterial diseases (MSMD) was first described in 1951.[119] The patients are susceptible to Bacillus Calmette-Guérin (BCG) vaccine substrains and environmental mycobacteria. Defects of 22 genes, underlying 47 allelic forms, with autosomal-recessive (AR), X-linked recessive (XLR), and autosomal-dominant (AD) modes of inheritance have been described.[120–126] The causal genes are physiologically related, as almost all their products are involved in interferon-γ (IFN-γ)-mediated immunity. Rare IEIs identified as causal for MSMD have been found in patients without MSMD but with TB as their sole phenotype.[127] Two disorders in particular have been diagnosed in several patients with TB, AR complete IL-12Rβ1 and TYK2 deficiencies, both of which impair both IL-12- and IL-23-dependent IFN-γ immunity. As these two disorders are rare, with a frequency of less than 10^{-5} in the general population, they account for only a very small proportion of TB cases even though their penetrance for TB is higher than that for MSMD, *M. tuberculosis* being about 1,000-fold more virulent than BCG and environmental mycobacteria. Nevertheless, this has provided proof of principle that defects of IFN-γ-mediated immunity can underlie isolated TB in humans without MSMD.[128–130]

These initial findings led to the discovery that homozygosity for the common p.Pro1104Ala (rs34536443) variant of *TYK2* underlies TB in patients of European ancestry.[67] Homozygosity for p.Pro1104Ala also underlies MSMD but with a much lower penetrance than for TB. This variant impairs the IL-23 response pathway as profoundly as complete deficiency of TYK2, but selectively, resulting in impairment of IFN-γ production by specific lymphocyte subsets without any detectable impact on the IL-12 response pathway (or the IL-10 and type I IFN pathways).[130,131] The frequency of this variant in Europeans is 0.0445, leading to a prevalence of about ~1 in 600 for homozygosity (Table 1). This variant is absent in sub-Saharan Africa and very rare in East Asia and has a prevalence of about 1% in other regions. In a subsequent study focusing on a European population based on UK Biobank data,[132] homozygosity for p.Pro1104Ala was found to account for about 1% of cases of TB in British individuals, with an OR of developing TB of 5 in homozygous carriers relative to heterozygotes or non-carriers.[68] Lifetime penetrance for the development of TB upon infection was estimated at about 80% for homozygotes (Table 2). Remarkably, the p.Pro1104Ala allele was also shown to have a protective effect against two autoimmune diseases, rheumatoid arthritis and systemic lupus erythematosus, possibly also accounting for its high frequency.[133] The p.Pro1104Ala allele originates from a founder effect about 30,000 years ago in Western Eurasians.[134] This accounts for its high prevalence in Europe, its presence in populations with European admixture, its rarity in Eastern Asia, and its absence from sub-Saharan Africa. The frequency of this variant has decreased slowly but steadily in Europeans over the last 2,000 years, from 13% to 4%, implying that negative selection has occurred, consistent with the very high burden of TB in Europe.[134] About one billion Europeans are estimated to have died from TB in the last 2,000 years.[68,135,136] Thus, studies of MSMD led to the discovery of a common monogenic etiology accounting for about 1% of past and present cases of TB in humans of European descent.

Common *IFNAR1* or *IFNAR2* variants can underlie viral diseases

Family-based studies of type I IFNs, a group of 16 IFNs (13 IFN-α genes, 2 of which encode identical proteins, IFN-β, IFN-ε, IFN-κ, and IFN-ω) binding a heterodimeric receptor composed of Interferon Alpha And Beta Receptor Subunit 1 (IFNAR1) and 2 (IFNAR2), have surprisingly led to discoveries of public health relevance.[137] Type I IFNs were first described in 1957 as molecules able to interfere with viral replication *in vitro*.[138] Upon binding to their receptor, type I IFNs induce a complex signaling cascade that plays a crucial role in antiviral immunity. IEIs impairing the type I IFN pathway underlie susceptibility to a narrow range of severe viral infections, including critical influenza pneumonia (e.g., IRF7, IRF9, and STAT2 deficiencies), critical COVID-19 pneumonia (e.g., IRF7, IFNAR1, IFNAR2, STAT2, and TYK2 deficiencies), herpes simplex virus 1 (HSV-I) encephalitis (e.g., IFNAR1), recurrent rhinovirus infection (e.g., IFIH1), and infections with live attenuated virus vaccines (e.g., IFNAR1, STAT1, and STAT2).[130,139–156] While the penetrance of individual viral infections is incomplete, most if not all patients suffer from at least one viral infection (Table 2). Autoantibodies against type I IFNs phenocopy IEIs of the IFN type I signaling pathway, further confirming the link between this pathway and sporadic severe viral infections.[152,157–159] Surprisingly, high frequencies of null alleles of *IFNAR1* and *IFNAR2* were recently reported in two geographically distant and isolated populations: Western Polynesians and Arctic people, respectively.[55,56,160] The p.Glu386* variant (rs201609461) was found with an allele frequency of 0.0125 in Western Polynesians, with a frequency of homozygosity estimated at ~1 in 6,500 in Samoans. The p.Ser53Pro variant (rs1987287426) of IFNAR2 is loss of function and was found with an allele frequency of 0.034 in Inuits from Greenland, Canada, and Alaska, with a homozygosity rate of ~1 in 1,500 Greenlanders. Homozygotes are prone to a small number of life-threatening viral illnesses. We cannot exclude the possibility that these variants provided a selective advantage to these populations in an unknown situation. However, in such geographically isolated populations, it appears more likely that this high allele frequency results from genetic drift with serial founder effects, isolation, or bottlenecks followed by rapid expansions of the population.[160,161]

Eleven hypomorphic IFNAR1 variants were recently identified.[53] These variants severely impair IFN-α and IFN-ω signaling but largely spare IFN-β signaling. Ten of these alleles are rare in all populations studied, but the remaining allele (p.Pro335del) is common in South Han Chinese, with an allele frequency ~0.02. As a result, it is predicted to be present in the homozygous state in ~1 of 2,500 individuals in this area[54] (Table 1). Homozygosity for the p.Pro335del allele was found to be associated with critical COVID-19 pneumonia in a 16-year-old patient. In addition, the hypomorphic variants show signs of negative dominance when co-expressed with the wild-type allele. Cells heterozygous for these variants display a dominant phenotype *in vitro*, with impaired responses to IFN-α and -ω but not -β, and viral

susceptibility. Consistent with these results, preliminary observations suggest that patients heterozygous for these variants are prone to respiratory and cerebral viral diseases with incomplete penetrance, attesting to both the dominance of these variants clinically and the importance of IFN-α and -ω for protective immunity against some respiratory and cerebral viruses. It is remarkable that a loss-of-function and dominant negative variant of IFNAR1 can reach such a high allele frequency. These results strongly suggest that the p.Pro335del variant is an important risk factor for severe viral infections in the South Han Chinese, at least when present in the homozygous state. With an estimated 16 million Chinese heterozygous for this variant, further population genetics studies are warranted to confirm the association of this variant with a higher risk of severe viral infections and estimate the clinical penetrance in both heterozygotes and homozygotes.

PATHOGENIC ALLELES WITH HIGH RISK BUT LOW PENETRANCE

Common variants at *HLA* loci underlie autoimmune conditions

Several human leukocyte antigen (HLA) alleles, some rare and some common, have been strongly associated with autoimmune conditions in population-based studies, conferring high risks with incomplete penetrance, with an OR >10 or even >50. These HLA-associated conditions may have been the evolutionary price to pay for the pathogen-driven positive selection of highly diverse HLA alleles over thousands of years.[162–164] As a trade-off, protection against infection early on, before or during reproductive age, may convey a risk of autoimmunity later in life, during or after the reproductive period. Alternatively, protection against infections in a given environment may convey a risk of autoimmunity in another environment, whether due to human migration to another region or environment modification in the same region. Both hypotheses can account for the dual phenotypes of individual humans but are more likely to manifest in populations over successive generations. Regardless, the larger the number of HLA alleles, the greater the diversity of microbial T cell epitopes presented to T cells in the infected population, and the higher the likelihood of at least some infected children and young adults surviving. In turn, the diversity of HLA alleles increases the risk that any given population may contain pathogenic variants at non-HLA loci controlling T cell tolerance to self and unleashing the recognition by T cells of self-peptides presented by HLA, the mechanism underlying clinical autoimmunity, particularly in middle-aged and elderly adults but not exclusively in these groups. This is the classic explanation of HLA-associated autoimmunity.

For example, HLA-B*57 (MAF ∼14%) and HLA-B*27 (MAF ∼8%) provide strong protection against disease progression in HIV-1-infected Europeans (OR = 7 and 3.4, respectively).[165,166] They may have protected children against other infections in the past. However, HLA-B*27 is also strongly associated with a higher risk of developing both ankylosing spondylitis (AS) (OR = 46) and post-infectious "reactive" arthritis (OR > 30).[167–170] Remarkably, AS penetrance is only 1.2% in HLA-B*27^{+} Europeans, whereas it is 21% in the HLA-B*27^{+} relatives of HLA-B*27^{+} AS patients, highlighting the considerable impact of variants at other loci.[171] However, this risk is not restricted to adults, as two frequent haplotypes, DRB1*03-DQB1*0201 (DR3) or DRB1*04-DQB1*0302 (DR4), which have frequencies of 1%–30% in most human populations, have been known since the 1970s to be associated with a higher risk of developing a life-threatening condition of childhood type 1 diabetes (T1D) (OR = 3.6 and 8.4, respectively).[172–175] The highest risk is for DR3/DR4 individuals (genotype frequency in European controls ∼2% versus ∼30% in T1D; OR = 18).[176] The persistence of common HLA alleles underlying a life-threatening condition of childhood when present in the heterozygous state suggests that these alleles may have conferred a major protective advantage in the past, possibly in another environment; that their pathogenic impact is more recent; or that their frequency had been steadily declining until insulin therapy became available. Thus, both rare and common HLA alleles can underlie autoimmune conditions. The description of all of these conditions is beyond the scope of this review.[177–179]

Common *NOD2* variants underlie Crohn's disease

Population-based studies have shown Crohn's disease — a chronic inflammatory bowel disease (IBD) characterized by patchy intestinal inflammatory lesions in the gastrointestinal tract leading to chronic abdominal pain, diarrhea, obstruction, and/or perianal lesions, can have a monogenic origin.[180] The prevalence of this disease is highest in North America, Western and Northern Europe, and Oceania but is increasing in other parts of the world, suggesting a strong impact of environmental factors.[180] Genome-wide linkage analyses have identified three frequent variants of *NOD2* (*nucleotide-binding oligomerization domain-containing 2*) collectively associated with an OR for disease development between 10 and 44 in homozygous or compound heterozygous carriers relative to controls.[21–23,64,65] The OR is much lower for heterozygotes (OR = 2.6). These three variants are p.Arg675Trp (rs2066844, also known as p.Arg702Trp), p.Gly881Arg (rs2066845, also known as p.Gly908Arg), and p.Leu980Profs*2 (rs2066847, also known as p.Leu1007Profs*2), with allelic frequencies ranging between 0.02 and 0.05 in Europeans and Middle Eastern individuals (Table 1). Despite the very strong association in multiple studies, a study of the Danish general population showed that the clinical penetrance of Crohn's disease at 50 years of age in biallelic carriers remains low, at ∼1.5% in homozygotes (Table 2).[23,80,181]

NOD2 may play a major role in regulation of the intestinal microbiota. It activates the NF-κB pathway in myeloid cells and ileal Paneth cells by recognizing the muramyl dipeptide (MDP) of intracellular bacterial lipopolysaccharides (LPSs).[182] NOD2 activation induces production of various cytokines, chemokines, and antimicrobial peptides in a cell type-dependent manner.[182] In particular, Paneth cells synthesize and secrete various antimicrobial peptides or proteins into the intestinal lumen, including lysozyme, human α-defensins 5 and 6 (HD5 and HD6, respectively), and secreted phospholipase A2 (sPLA2). In the mouse model, bacterial killing by Nod2-deficient ileal cells is impaired, resulting in ileal dysbiosis. This dysbiosis increases the interaction of bacterial products with immune cells, leading to chronic inflammation and the typical histological presentation, with transmural infiltration by lymphocytes

and macrophages, together with granuloma. The three frequent variants associated with Crohn's disease are hypomorphic, as they retain their ability to induce basal NF-κB activation in the absence of activation, but completely fail to activate NF-κB signaling in the presence of LPSs.[21,65] It has been suggested that their high allelic frequency in Europeans attests to a protective role against septic shock in carriers, as demonstrated in the mouse model.[183]

A common *CCR5* variant protects against HIV and confers a predisposition to West Nile virus infection

Population-based human genetic studies have investigated infection with human immunodeficiency virus 1 (HIV-1), a retrovirus primarily infecting CD4$^+$ T cells, leading to their progressive loss and, ultimately, to acquired immunodeficiency syndrome (AIDS).[184] CD4 is the receptor for HIV-1 on CD4$^+$ T cells, with C-C chemokine receptor 5 (CCR5) as the principal coreceptor. Autosomal-recessive CCR5 deficiency confers resistance to infection with HIV-1 with high, if not complete, penetrance.[47–49] Certain protective *CCR5* alleles are rare (e.g., p.Cys101*, also known as c.303T>A or rs1800560),[185] but at least one loss-of-function allele of *CCR5* is common: a 32-bp deletion leading to premature termination of protein synthesis (rs333, p.Ser185Ilefs*32, CCR5-Δ32). This allele has a MAF of ∼0.1 in Europeans, among whom the rate of homozygosity is ∼1% (Table 1). The CCR5-Δ32 allele is less frequent, or even absent, in African and Asian populations. Given the recent emergence of HIV-1, resistance to this virus cannot explain the high frequency of the CCR5-Δ32 allele in the European population. This allele emerged in Northern Europe at least 7,000 years ago, and stabilized to its modern frequency around 2,000 years ago, suggesting earlier selection events.[186] It has been suggested that there was intensive selection for the CCR5-Δ32 variant during ancient pandemics of diseases such as plague or smallpox.[187–189]

CCR5-deficient individuals are otherwise apparently normal, but two studies have suggested that they have a higher risk of symptomatic West Nile virus (WNV) disease (WNVD), with an OR of 5.9 in Europeans (Table 2).[24,25,70] The penetrance is unknown. Consistently, CCR5-deficient mice invariably develop fatal WNV encephalitis upon infection.[190] WNV is an RNA flavivirus transmitted by bites from infected mosquitoes. Clinical manifestations occur in only 20% of infected individuals and include fever, headache, tiredness, body aches, nausea, vomiting, skin rash, and swollen lymph glands. Less than 1% of infected individuals develop life-threatening WNV encephalitis. Remarkably, autoantibodies neutralizing type 1 IFNs underlie about 40% of cases of WNV encephalitis.[158] It may not, therefore, be coincidental that CCR5 is highly expressed on T cells and plasmacytoid dendritic cells (pDCs), the most potent type I IFN-producing cell types. CCR5 deficiency may impair optimal recruitment of pDCs to the site of infection. This hypothesis is plausible, as it would be consistent with the risk conferred by auto-antibodies against type I IFNs and with patients with various inherited or acquired conditions of T cells not prone to WNVD. As WNV has only recently reached the shores of Southern Europe, predisposition to lethal disease in homozygotes is consistent with the spread of the mutant CCR5 allele from Northern to Southern Europe due to elusive selective forces.

A common variant of *TIM3* underlies SPTCL with a high risk of HLH

Subcutaneous panniculitis-like T cell lymphoma (SPTCL), accounting for less than 1% of diagnosed non-Hodgkin's lymphomas, is characterized by the infiltration of CD8$^+$ αβ T cells into the subcutaneous adipose tissue, where they surround adipocytes in a lace-like pattern.[191–193] Affected individuals typically display multiple subcutaneous nodules, night sweats, fever, and weight loss, and, in ∼20% of cases, associated autoimmune disorders, most commonly systemic lupus erythematosus.[194] In about 20% of cases, SPTCL is associated with hemophagocytic lymphohistiocytosis (HLH),[193] life-threatening hyperinflammation caused by uncontrolled activation of lymphocytes and macrophages. In their study of a series of 27 patients with SPTCL, Gayden et al. found that 60% of their patients carried germline biallelic loss-of-function variants of the hepatitis A virus cellular receptor 2 (*HAVCR2*) gene, which encodes T cell immunoglobulin mucin 3 (TIM3).[52] TIM3 is the third member of the TIM family. It is widely expressed across the leukocytes of the immune system. Its best characterized ligand is galectin-9, which is strongly expressed on myeloid cells and endothelial cells and in the gastrointestinal tract. Following binding, TIM3 acts as an immune checkpoint in the maintenance of self-tolerance and antitumoral immunity.[195,196] TIM-3-deficient SPTCL patients have an earlier age at onset of disease than other patients and an extremely high risk of developing HLH (30–80%).[52,197]

Intriguingly, most of the patients in the first series were from Polynesia and East Asia, and all carried the same variant, p.Tyr82Cys (rs184868814). Population genetics analysis revealed allele frequencies of 0.04 and 0.01656 (gnomAD v.3.1) in Polynesia and East Asia, respectively (Table 1).[52] These findings were replicated in other series.[197–199] Māori/Pacific individuals were shown to have a higher risk of SPTCL than Europeans (RR = 11),[72] whereas the OR in East Asian homozygotes was estimated at ∼10,000 relative to East Asian heterozygotes or non-carriers.[198] With 1 in 625 Polynesians and 1 in 3,600 East Asians predicted to be homozygous for the loss-of-function p.Tyr82Cys variant, TIM3 deficiency clearly drives SPTCL with low penetrance (if all SPTCL cases in Polynesians are attributable to TIM3 deficiency, then the maximum penetrance in p.Tyr82Cys homozygotes, based on SPTCL incidence in Māori/Pacific individuals,[72] would be 0.34%/year and 20.4% over 60 years) (Table 2). However, the strength of the association is beyond reasonable doubt, and the breadth of the associated phenotypes remains to be assessed in large series of carriers. The very high frequency of the p.Tyr82Cys variant in Polynesians probably results from bottlenecks followed by expansion, but the reasons for its high frequency in East Asians remains unclear.

Common *PTCRA* alleles can underlie autoimmunity

Patient-based studies of rare patients with pre-TCRα deficiency led to another observation of public health relevance. Adaptive immunity is defined by subsets of cells using rearranged antigen receptors, including αβ T cells, γδ T cells, and B cells. αβ and γδ T cells differentiate from bone marrow-derived progenitors in the thymus. Early thymocytes simultaneously rearrange their TCR δ, TCRγ and TCRβ loci. If early thymocytes successfully rearrange the TCRγ and TCRδ loci, then they differentiate into γδ T cells.

If they successfully rearrange the TCRβ, then the TCRβ chain is expressed at the cell surface in the pre-TCR complex thanks to its dimerization with the pre-TCRα constant chain (pTα; encoded by *PTCRA*). This process, known as β-selection, is essential for thymocyte survival and proliferation before TCRα rearrangement and differentiation into mature αβ T cells. Complete T cell differentiation defects are associated with SCID phenotypes (see common variants of *ADA*, *DCLRE1C*, and *IKBKB* can underlie severe T cell deficiencies), whereas partial T cell differentiation defects are associated with autoimmunity and a less severe susceptibility to infection. Mice lacking pTα have >95% fewer αβ T cells in the periphery. Ten humans homozygous or compound heterozygous for private or rare biallelic loss-of-function variants leading to complete pTα deficiency and impaired pre-TCR complex formation were recently reported.[66] These patients have a small thymus, profound αβ T cell lymphopenia in early life, and abnormally high counts of γδ T cells. Despite this severe biological phenotype, they remain asymptomatic until their teenage years or early adulthood and develop infections and autoimmunity. This late disease onset can be explained by the production of a small but sufficient number of functional αβ T cells providing protection against infectious diseases.

In the same study,[66] two hypomorphic *PTCRA* alleles present in the general population were also identified: p.Tyr76Cys (rs141630791) and p.Asp51Ala (rs200942121). Both alleles severely impair pre-TCR complex formation *in vitro*. The p.Tyr76Cys allele is found in people of African ancestry, with an allele frequency of 0.003517 (gnomAD v.4.1), and is predicted to be present in the homozygous state in 1 in 80,000 Africans, possibly more in specific African populations. The p.Asp51Ala variant is common in the Middle East and South Asia, with an allele frequency as high as 0.011 in Iran and 0.020 in Pakistan,[66] and is therefore predicted to be homozygous in 1 in 2,500–10,000 individuals in the corresponding populations (Table 1). Like mice with similar knockin mutations,[200] homozygous p.Asp51Ala carriers produce abnormally large numbers of γδ naive T cells, but their peripheral αβ T cell counts are normal.[66] In a large cohort, p.Asp51Ala homozygotes had a five times higher risk of developing autoimmunity than heterozygotes or non-carriers, with a disease penetrance of about 12% (Table 2). However, this risk and penetrance are probably underestimated because the cohort studied was young (mean age: 9.5 years), and the cumulative risk of developing autoimmunity increases with age. This variant may have provided a selective advantage in a specific, past environment. Alternatively, it may not be purged because it does not significantly decrease survival fitness before reproductive age, and even then only in homozygotes.

DISCUSSION

Monogenic immunological disorders involving common variants have already been identified for 15 human loci (with *HLA* arbitrarily considered as a single locus). These genotypes were discovered in patient-based studies of rare (e.g., FMF) or common (e.g., TB) conditions (*MEFV*, *C2*, *C6*, *C9*, *DCLRE1C*, *ADA*, *IKBKB*, *TYK2*, *TIM3*, *IFNAR1*, *IFNAR2*, and *PTCRA*) and population-based studies of common conditions (*HLA*, *NOD2*, and *CCR5*). After a pause between 2013 and 2018, their rate of discovery has recently accelerated (Figure 2). The first five common alleles underlying monogenic immunological disorders were identified in the last quarter of the 20th century (*HLA*, *MEFV*, *C2*, *C6*, and *C9*), another five were identified between 2000 and 2013 (*NOD2*, *DCLRE1C*, *ADA*, *CCR5*, and *IKBKB*), and the remaining five (*TYK2*, *TIM3*, *IFNAR1*, *IFNAR2*, and *PTCRA*) have been discovered since 2018. Remarkably, some of these genes carry several common variants that are pathogenic (*HLA*, *MEFV*, *NOD2*, and *IFNAR1*). Some of these alleles are common only in small, isolated populations, as best exemplified by a null *IKBKB* allele underlying SCID in homozygotes of Northern Cree decent.

By contrast, some of the other alleles are common in large populations, as illustrated by a severely hypomorphic *PTCRA* allele in the Middle East and South Asia, where homozygotes are prone to various types of autoimmunity. Most of these variants are recessive, causing disease only when present in the homozygous state. The frequency of at-risk genotype carriers (homozygous and compound heterozygous) is therefore much lower than the MAF. Admittedly, the MAF cutoff used to separate rare and common alleles (0.01) is arbitrary, with no significant genetic difference between a MAF of 0.009 and a MAF of 0.011. The definition of a population is also arbitrary, ranging from a hamlet to one of the seven major ancestries. Moreover, only a small proportion of populations of intermediate size and an even smaller proportion of smaller populations have been subject to sufficiently profound genetic analyses to estimate the most relevant MAF (i.e., the MAF of the allele in the smallest relevant population) with a reasonable degree of confidence. It therefore seems plausible that there are many more common alleles underlying monogenic immunological conditions.

Excluding *HLA*, which merits its own separate analysis, we identified 17 common variants at 13 loci. This number is relatively small next to the >450 monogenic IEIs due to rare variants discovered in patient-based studies. This probably results from the common practice of filtering out common alleles when searching for new IEIs – and monogenic inborn errors at large. We think that this notion has important implications for future research in biology and medicine. Some population-based studies originally detected common variants of single genes as disease causing (*HLA*, *NOD2*, and *CCR5*), but only a few have tested the hypothesis of recessive inheritance. This aspect should probably be reconsidered, particularly for very large studies with high statistical power. This notion also has important implications for patient-based studies. These findings suggest that, during genetic study of a patient or group of patients, the population to which they belong should be defined as accurately as possible. For example, three unrelated patients from France, Germany, and Italy would have Western Europe as a common denominator. The populations of the corresponding countries should also serve individually as reference populations. Use of the province of origin (e.g., Brittany, Piedmont, and Bavaria) would provide even higher granularity. The MAF of candidate alleles and the prevalence of the phenotype of interest should be defined at these different levels. The genetic data for each patient should ideally be considered in the context of their smallest, relevant population. The MAF of an allele in the "human

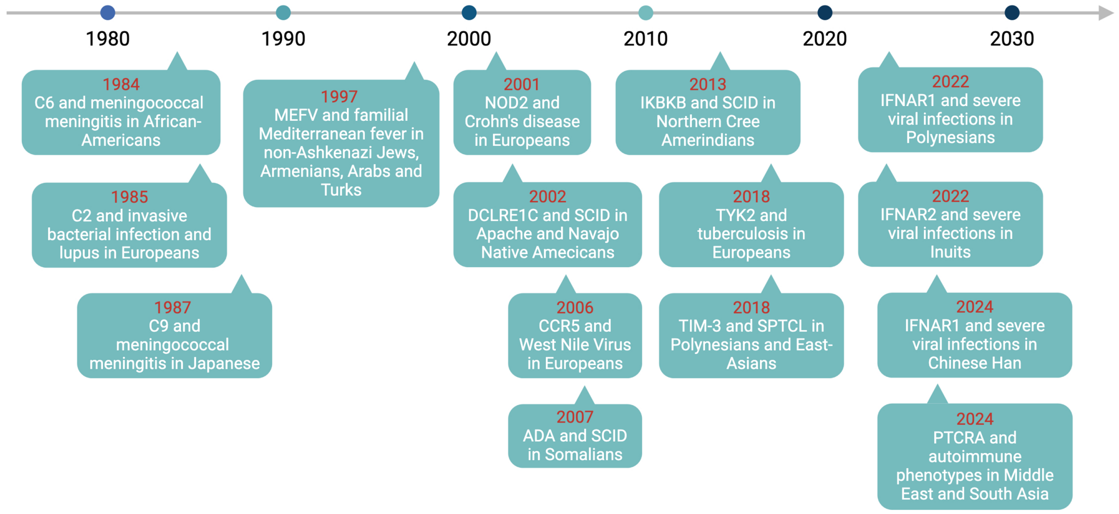

Figure 2. Timeline of the discovery of common IEIs to infections

We indicate the time point when the common molecular defect was associated with the indicated phenotype. Some of the molecular or immunological defects were known for decades before the association between the genotype and the phenotype was established.

ADA: ADA deficiency was identified in two sporadic SCID cases in 1972,[201] the first mutations in ADA were reported in 1986,[202] the first Somalian patient with the p.Gln3* variant was identified in 1995,[114] and the high prevalence of partial ADA deficiency in Somalians was recognized in 2007.[39]

C2: C2 deficiency was identified in sporadic cases in 1966,[33] the high prevalence of complete C2 deficiency in Europeans was reported in 1985,[34] and the corresponding variant was discovered in 1992.[35]

C6: C6 deficiency was identified in sporadic cases in 1974,[87] the high prevalence of complete C6 deficiency in Afro-Americans was reported in 1984,[41] and the corresponding variant was discovered in 1996.[42]

C9: C9 deficiency was identified in sporadic cases in 1979,[92] the high prevalence of complete C9 deficiency in the Japanese was reported in 1987[93] and its association with meningococcal diseases in 1989,[43] and the corresponding variant was identified in 1998.[94]

CCR5: CCR5 deficiency and its relationship to resistance to HIV-1 infection were discovered simultaneously in 1996.[47–49] The higher risk of developing symptomatic WNV infection in CCR5-deficient individuals was discovered in 2006.[24]

DCLRE1C (ARTEMIS): The high prevalence of SCID in Apache and Navajo populations was first described in 1980.[50] The prevalence of heterozygous carriers in these populations was estimated at 2.1% in 1991.[112] The causal mutation of *DCLRE1C* was identified in 2002.[51]

HAVCR2 (TIM-3): complete TIM-3 deficiency and its association with SPTCL and HLH were described together in 2018.[52]

IFNAR1: complete IFNAR1 deficiency and its association with severe viral infections were discovered in 2019.[142] The discovery of a frequent loss-of-function variant in Polynesians was reported in 2022.[55] A frequent hypomorphic variant in South Chinese Han was reported in 2024.[53]

IFNAR2: complete IFNAR2 deficiency and its association with severe viral infections were discovered in 2015.[140] A frequent loss-of-function variant in Inuits was reported in 2022.[56]

IKBKB: A high prevalence of IKBKB deficiency was detected in the Northern Cree in 2013, corresponding to the first description of human IKBKB deficiency.[57] This observation was later confirmed by newborn screening.[58]

MEFV: familial Mediterranean fever (FMF) was first described in 1908.[96] The high prevalence of FMF in Jewish individuals and Armenians was reported in 1945.[97] The corresponding frequent variants of the *MEFV* gene were identified 1997.[59,60]

NOD2: NOD2 deficiency and its association with Crohn's disease were described together in 2001.[21–23]

PTCRA: the hypomorphic PTCRA variant frequent in South Asia and the Middle East was identified together with the rare complete loss-of-function variants.[66] The frequent hypomorphic variant is associated with autoimmune phenotypes with incomplete penetrance.

TYK2: complete TYK2 deficiency was first described in 2006[203] and its association with viral and mycobacterial diseases in 2015.[129] A common variant underlying partial TYK2 deficiency and susceptibility to TB in Europeans was discovered in 2018.[67]

general population" is not informative enough for rigorous analysis of a patient's exome or genome.

In this light, we think that common alleles should not be systematically filtered out when considering phenotypes that have a local or global prevalence between 10^{-4} and 10^{-2} for recessive traits and even perhaps 10^{-5}. This emphasizes the importance of ascertaining population prevalence of a given immunological disorder, which should match the MAF of fully penetrant pathogenic alleles (e.g., SCID in Navajo and Apache populations). Nevertheless, incomplete penetrance should be considered (Table 2), which may account for an apparent mismatch between disease prevalence and MAF of pathogenic alleles. In total, 14 common alleles are currently known to be autosomal and to underlie recessive phenotypes, and another three are known to be semi-dominant (two variants of *MEFV* and one of *IFNAR1*) (Table 1). However, AD and XLR modes of inheritance should also be considered. For XLR and AD disorders, phenotypes with a higher prevalence, up to 10^{-2}, can be considered.

The evolutionary basis of the surprising commonality of the variants described in this review is mostly hypothetical and probably varies from case to case. For instance, population bottlenecks followed by rapid expansion are the most likely hypothesis explaining the high frequency of SCID-causing *IKBKB* or *DCLRE1C* variants in Native American populations. A similar mechanism probably also explains the high frequency of null *IFNAR1* and *IFNAR2* alleles in Polynesians and Arctic people,

respectively. The reasons for the high frequency of a severely hypomorphic and dominant-negative *IFNAR1* allele in Southern Chinese are less clear. Recessive alleles are counterselected only in individuals carrying two deleterious copies and may therefore persist at high frequencies for longer periods of time, particularly if they display incomplete penetrance or are associated with a mild clinical phenotype. This is probably the case for the p.Pro1104Ala *TYK2* allele, whose the frequency has declined in Europeans from 13% to 4%, over the last 3,000 years due to the selective pressure exerted by TB on homozygotes.

A third mechanism is balancing selection due to beneficial or pathogenic properties of the variant depending on zygosity. The best example is perhaps provided by variants of *MEFV* for which heterozygosity is thought to protect against plague, whereas homozygosity underlies FMF. Likewise, C2, C6, and C9 deficiencies confer predisposition to invasive bacterial infections, whereas the variants concerned may protect against septic shock in heterozygotes. In addition, the same genotype may have opposite effects, depending on the microbial environment, which itself varies over time and space. For example, homozygosity for variants causing CCR5 deficiency protects against HIV-1 infection and may have protected against other unknown pathogens in the past, but it also confers a predisposition to WNVD. Likewise, variants of *NOD2*, *PTCRA*, *TYK2*, or *TIM3* may have become common due to genetic drift before the advent of recent environmental triggers that revealed their pathogenicity. For instance, the recent rapid, global increase in the prevalence of Crohn's disease in homozygotes for pathogenic *NOD2* variants may be due to the recent spread of ad hoc environmental cues.

These findings also have practical implications. When a new monogenic disorder due to rare alleles is discovered, systematic experimental studies of the other alleles at the same locus should be performed. This is important not only to ensure that the cumulative frequency of the deleterious genotype, whether loss of function or gain of function, is consistent with the prevalence of the phenotype studied but also because it may lead to the discovery that the same genotype and phenotype are less rare, and perhaps even common, in another small or even large population. Moreover, this approach may reveal that common variants have different biochemical impacts, raising the possibility that they underlie another phenotype that may (e.g., if common alleles are hypomorphic and the rare alleles are loss of function) or may not (e.g., if common alleles are gain of function [GoF] or neomorphic and the rare alleles are loss-of-function) be related.

The *PTCRA* gene neatly illustrates this point, as the discovery of ultra-rare alleles underlying complete deficiency of pre-TCRα in homozygotes with overt clinical immunodeficiency led to the discovery of an almost complete deficiency in hundreds of thousands of South Asians and Middle Eastern individuals with various isolated forms of autoimmunity and only a mild immunological phenotype. Before the advent of whole-exome and whole-genome sequencing, the search for IEIs only rarely took into account the MAF of candidate alleles, let alone the cumulative MAFs of the deleterious alleles of a gene. Sequencing of 50 or 100 healthy controls, rarely of homogenous or relevant ancestry, was often deemed sufficient. Nowadays, thanks to large public databases (e.g., gnomAD),[36] it is easy to obtain access to information about rare and common alleles in all major ancestries and even increasingly in more specific populations. Experimental testing of all alleles has become a requirement, with discovery of common and deleterious alleles revealing the breadth of consequences of monogenic lesions in multiple populations.

Finally, these findings imply that monogenic lesions should be considered in patients with clinical phenotypes that are not as rare as those typically associated with IEIs and that such lesions can be discovered not only in population-based studies but also in patient-based studies that are extended into population-based studies. A significant proportion of cases of "common diseases" may be caused by monogenic IEIs and common alleles. This is neatly illustrated by the *PTCRA* alleles underlying autoimmunity and the *TYK2* allele underlying TB, both of which highlight the importance of considering common alleles for recessive traits, not just, as traditionally, for dominant or semidominant (additive) traits.

The genetic architecture of common diseases may thus be revisited by considering monogenic common lesions. Recessive traits should be considered in genome-wide association studies (GWASs) of common variants; they may be highly penetrant in a subset of or the entire population sample. Candidate monogenic genotypes may then be investigated experimentally through targeted functional and familial genetic studies. Moreover, statistical significance does not equal biological significance; the TYK2 p.Pro1104Ala variant is not statistically significant when tested by GWAS for association with TB in a recessive model because of the correction for multiple testing. However, it is clearly causal when computational and experimental lines of evidence are considered together. The limitations imposed by the need to correct for multiple testing and by the experimental difficulties to test non-coding common variants are intrinsic to the GWAS approach. Common immunological diseases may be due to various recessive or dominant traits involving common alleles in a significant proportion of patients. Based on these findings and this model, it is likely that many more patients worldwide than previously thought suffer from monogenic disorders. The findings described in this review suggest that there may be millions of people with monogenic immunological disorders. They suggest that forward genetic approaches may not be sufficient to appreciate fully the impact of single-gene lesions. Reverse genetic approaches are probably warranted for experimental testing of all alleles at specific loci before computational and agnostic assessments of the clinical impact of biochemically deleterious alleles in different populations.

ACKNOWLEDGMENTS

We would like to thank all members of the HGID laboratory for fruitful discussions, particularly Laurent Abel, Aurélie Cobat, and Jérémie Rosain for critical reading of an earlier version of this paper. This work was supported in part by the St. Giles Foundation; the Rockefeller University; Institut National de la Santé et de la Recherche Médicale (INSERM); the Imagine Institute; Paris Cité University; the National Center for Research Resources; the National Center for Advancing Sciences of the National Institutes of Health (NIH) (UL1TR001866); the NIH (R01AI088364, R01AI095983, R01AI163029, and U19AI162568); the American Lung Association (COVID-1026207); the Stavros

Niarchos Foundation (SNF) as part of its grant to the SNF Institute for Global Infectious Disease Research at The Rockefeller University; the Square Foundation, Grandir – Fonds de solidarité pour l'enfance; the Fondation du Souffle; the SCOR Corporate Foundation for Science; the Battersea and Bowery Advisory Group; the French National Research Agency (ANR) under the "Investments for the Future" program (ANR-10-IAHU-01); the Integrative Biology of Emerging Infectious Diseases Laboratory of Excellence (ANR-10-LABX-62-IBEID); ANR GENVIR (ANR-20-CE93-003); ANR AAILC (ANR-21-LIBA-0002); ANR AI2D (ANR-22-CE15-0046); ANR MAFMACRO (ANR-22-CE92-0008); ANR GENFLU (ANR-22-CE92-0004); ANR PTCRA (ANR-24-CE15-5334); the ANR-RHU COVIFERON program (ANR-21-RHUS-08); the French research agency on infectious and emerging diseases (ANRS) project ECTZ170784-ANRS0073; the Horizon-HLTH-2021-DISEASE-04 program under grant agreement 101057100 (UNDINE); the European Union's Horizon 2020 Research and Innovation Program under grant agreement 824110 (EASI-genomics); the French Foundation for Medical Research (Equation 201903007798); the French foundation for cancer research (ARC) project AR-CAGEING2022040004944 and ARCPGA2024110008994_9650, Robert Debré Association for Medical Research, W.E. Ford, General Atlantic's Chairman and Chief Executive Officer, G. Caillaux, General Atlantic's Co-President, Managing Director, and Head of Business in EMEA, and the General Atlantic Foundation; the French Ministry of Higher Education, Research, and Innovation (MESRI-COVID-19); and REACTing-INSERM.

DECLARATION OF INTERESTS

The authors declare no competing interests.

SUPPLEMENTAL INFORMATION

Supplemental information can be found online at https://doi.org/10.1016/j.xgen.2025.101129.

REFERENCES

1. Lutz, W. (1946). A propos de l'Epidermodysplasie verruciforme. Dermatologica (Basel) *92*, 30–43.
2. Kostmann, R. (1950). Hereditär reticulos-en ny systemsjukdom. Svenska Läkartideningen *47*, 2861–2868.
3. Bruton, O.C. (1952). Agammaglobulinemia. Pediatrics *9*, 722–728.
4. Notarangelo, L.D., Bacchetta, R., Casanova, J.-L., and Su, H.C. (2020). Human inborn errors of immunity: An expanding universe. Sci. Immunol. *5*, eabb1662.
5. Gruber, C., and Bogunovic, D. (2020). Incomplete penetrance in primary immunodeficiency: a skeleton in the closet. Hum. Genet. *139*, 745–757.
6. Israel, L., Wang, Y., Bulek, K., Della Mina, E., Zhang, Z., Pedergnana, V., Chrabieh, M., Lemmens, N.A., Sancho-Shimizu, V., Descatoire, M., et al. (2017). Human Adaptive Immunity Rescues an Inborn Error of Innate Immunity. Cell *168*, 789–800.e10.
7. Spaan, A.N., Neehus, A.L., Laplantine, E., Staels, F., Ogishi, M., Seeleuthner, Y., Rapaport, F., Lacey, K.A., Van Nieuwenhove, E., Chrabieh, M., et al. (2022). Human OTULIN haploinsufficiency impairs cell-intrinsic immunity to staphylococcal α-toxin. Science *376*, eabm6380.
8. Alcaïs, A., Quintana-Murci, L., Thaler, D.S., Schurr, E., Abel, L., and Casanova, J.L. (2010). Life-threatening infectious diseases of childhood: single-gene inborn errors of immunity? Ann. N. Y. Acad. Sci. *1214*, 18–33.
9. Fieschi, C., Dupuis, S., Catherinot, E., Feinberg, J., Bustamante, J., Breiman, A., Altare, F., Baretto, R., Le Deist, F., Kayal, S., et al. (2003). Low penetrance, broad resistance, and favorable outcome of interleukin 12 receptor beta1 deficiency: medical and immunological implications. J. Exp. Med. *197*, 527–535.
10. Stewart, O., Gruber, C., Randolph, H.E., Patel, R., Ramba, M., Calzoni, E., Huang, L.H., Levy, J., Buta, S., Lee, A., et al. (2025). Monoallelic expression can govern penetrance of inborn errors of immunity. Nature *637*, 1186–1197. https://doi.org/10.1038/s41586-024-08346-4.
11. Hirschhorn, R., Yang, D.R., Puck, J.M., Huie, M.L., Jiang, C.K., and Kurlandsky, L.E. (1996). Spontaneous in vivo reversion to normal of an inherited mutation in a patient with adenosine deaminase deficiency. Nat. Genet. *13*, 290–295.
12. Timberlake, A.T., Choi, J., Zaidi, S., Lu, Q., Nelson-Williams, C., Brooks, E.D., Bilguvar, K., Tikhonova, I., Mane, S., Yang, J.F., et al. (2016). Two locus inheritance of non-syndromic midline craniosynostosis via rare SMAD6 and common BMP2 alleles. eLife *5*, e20125.
13. Simchoni, N., Koide, S., Likhite, M., Kuchitsu, Y., Kadirvel, S., Law, C.S., Elicker, B.M., Kurra, S., Wong, M.M.K., Yuan, B., et al. (2025). The common HAQ STING allele prevents clinical penetrance of COPA syndrome. J. Exp. Med. *222*, e20242179.
14. Bergson, S., Sarig, O., Giladi, M., Mohamad, J., Mogezel-Salem, M., Smorodinsky-Atias, K., Sade, O., Manori, B., Assaf, S., Malovitski, K., et al. (2025). HMCN1 variants aggravate epidermolysis bullosa simplex phenotype. J. Exp. Med. *222*, e20240827.
15. Uda, M., Galanello, R., Sanna, S., Lettre, G., Sankaran, V.G., Chen, W., Usala, G., Busonero, F., Maschio, A., Albai, G., et al. (2008). Genome-wide association study shows BCL11A associated with persistent fetal hemoglobin and amelioration of the phenotype of β-thalassemia. Proc. Natl. Acad. Sci. USA *105*, 1620–1625.
16. McGee, T.L., Devoto, M., Ott, J., Berson, E.L., and Dryja, T.P. (1997). Evidence That the Penetrance of Mutations at the RP11 Locus Causing Dominant Retinitis Pigmentosa Is Influenced by a Gene Linked to the Homologous RP11 Allele. Am. J. Hum. Genet. *61*, 1059–1066.
17. Castel, S.E., Cervera, A., Mohammadi, P., Aguet, F., Reverter, F., Wolman, A., Guigo, R., Iossifov, I., Vasileva, A., and Lappalainen, T. (2018). Modified penetrance of coding variants by cis-regulatory variation contributes to disease risk. Nat. Genet. *50*, 1327–1334.
18. Zhu, H., Zhang, J., Rao, S., Durbin, M.D., Li, Y., Lang, R., Liu, J., Xiao, B., Shan, H., Meng, Z., et al. (2025). Common cis-regulatory variation modifies the penetrance of pathogenic SHROOM3 variants in craniofacial microsomia. Genome Res. *35*, 1065–1079.
19. Casanova, J.-L., Conley, M.E., Seligman, S.J., Abel, L., and Notarangelo, L.D. (2014). Guidelines for genetic studies in single patients: lessons from primary immunodeficiencies. J. Exp. Med. *211*, 2137–2149.
20. Forrest, I.S., Chaudhary, K., Vy, H.M.T., Petrazzini, B.O., Bafna, S., Jordan, D.M., Rocheleau, G., Loos, R.J.F., Nadkarni, G.N., Cho, J.H., and Do, R. (2022). Population-Based Penetrance of Deleterious Clinical Variants. JAMA *327*, 350–359.
21. Ogura, Y., Bonen, D.K., Inohara, N., Nicolae, D.L., Chen, F.F., Ramos, R., Britton, H., Moran, T., Karaliuskas, R., Duerr, R.H., et al. (2001). A frameshift mutation in NOD2 associated with susceptibility to Crohn's disease. Nature *411*, 603–606.
22. Hugot, J.-P., Chamaillard, M., Zouali, H., Lesage, S., Cézard, J.P., Belaiche, J., Almer, S., Tysk, C., O'Morain, C.A., Gassull, M., et al. (2001). Association of NOD2 leucine-rich repeat variants with susceptibility to Crohn's disease. Nature *411*, 599–603.
23. Hampe, J., Cuthbert, A., Croucher, P.J., Mirza, M.M., Mascheretti, S., Fisher, S., Frenzel, H., King, K., Hasselmeyer, A., MacPherson, A.J., et al. (2001). Association between insertion mutation in *NOD2* gene and Crohn's disease in German and British populations. Lancet *357*, 1925–1928.
24. Glass, W.G., McDermott, D.H., Lim, J.K., Lekhong, S., Yu, S.F., Frank, W.A., Pape, J., Cheshier, R.C., and Murphy, P.M. (2006). CCR5 deficiency increases risk of symptomatic West Nile virus infection. J. Exp. Med. *203*, 35–40.
25. Lim, J.K., McDermott, D.H., Lisco, A., Foster, G.A., Krysztof, D., Follmann, D., Stramer, S.L., and Murphy, P.M. (2010). CCR5 Deficiency Is a Risk Factor for Early Clinical Manifestations of West Nile Virus Infection but not for Viral Transmission. J. Infect. Dis. *201*, 178–185.

26. Bonthron, D.T., Markham, A.F., Ginsburg, D., and Orkin, S.H. (1985). Identification of a point mutation in the adenosine deaminase gene responsible for immunodeficiency. J. Clin. Investig. *76*, 894–897.
27. Royer-Pokora, B., Kunkel, L.M., Monaco, A.P., Goff, S.C., Newburger, P.E., Baehner, R.L., Cole, F.S., Curnutte, J.T., and Orkin, S.H. (1986). Cloning the gene for an inherited human disorder—chronic granulomatous disease—on the basis of its chromosomal location. Nature *322*, 32–38.
28. Mold, C. (1999). Role of complement in host defense against bacterial infection. Microbes Infect. *1*, 633–638.
29. Dunkelberger, J.R., and Song, W.-C. (2010). Complement and its role in innate and adaptive immune responses. Cell Res. *20*, 34–50.
30. Casanova, J.L., and Abel, L. (2004). Human Mannose-binding Lectin in Immunity: Friend, Foe, or Both? J. Exp. Med. *199*, 1295–1299.
31. Leffler, J., Bengtsson, A.A., and Blom, A.M. (2014). The complement system in systemic lupus erythematosus: an update. Ann. Rheum. Dis. *73*, 1601–1606.
32. Truedsson, L. (2015). Classical pathway deficiencies – A short analytical review. Mol. Immunol. *68*, 14–19.
33. Klemperer, M.R., Woodworth, H.C., Rosen, F.S., and Austen, K.F. (1966). Hereditary deficiency of the second component of complement (C'2) in man. J. Clin. Investig. *45*, 880–890.
34. Cole, F.S., Whitehead, A.S., Auerbach, H.S., Lint, T., Zeitz, H.J., Kilbridge, P., and Colten, H.R. (1985). The Molecular Basis for Genetic Deficiency of the Second Component of Human Complement. N. Engl. J. Med. *313*, 11–16.
35. Johnson, C.A., Densen, P., Hurford, R.K., Colten, H.R., and Wetsel, R.A. (1992). Type I human complement C2 deficiency. A 28-base pair gene deletion causes skipping of exon 6 during RNA splicing. J. Biol. Chem. *267*, 9347–9353.
36. Chen, S., Francioli, L.C., Goodrich, J.K., Collins, R.L., Kanai, M., Wang, Q., Alföldi, J., Watts, N.A., Vittal, C., Gauthier, L.D., et al. (2024). A genomic mutational constraint map using variation in 76,156 human genomes. Nature *625*, 92–100.
37. Pickering, M.C., Botto, M., Taylor, P.R., Lachmann, P.J., and Walport, M.J. (2000). Systemic Lupus Erythematosus, Complement Deficiency, and Apoptosis. Adv. Immunol. *76*, 227–324.
38. Jönsson, G., Truedsson, L., Sturfelt, G., Oxelius, V.A., Braconier, J.H., and Sjöholm, A.G. (2005). Hereditary C2 Deficiency in Sweden: Frequent Occurrence of Invasive Infection, Atherosclerosis, and Rheumatic Disease. Medicine *84*, 23–34.
39. Sanchez, J.J., Monaghan, G., Børsting, C., Norbury, G., Morling, N., and Gaspar, H.B. (2007). Carrier Frequency of a Nonsense Mutation in the Adenosine Deaminase (ADA) Gene Implies a High Incidence of ADA-deficient Severe Combined Immunodeficiency (SCID) in Somalia and a Single, Common Haplotype Indicates Common Ancestry. Ann. Hum. Genet. *71*, 336–347.
40. Adams, S.P., Wilson, M., Harb, E., Fairbanks, L., Xu-Bayford, J., Brown, L., Kearney, L., Madkaikar, M., and Bobby Gaspar, H. (2015). Spectrum of mutations in a cohort of UK patients with ADA deficient SCID: Segregation of genotypes with specific ethnicities. Clin. Immunol. *161*, 174–179.
41. Ross, S.C., and Densen, P. (1984). Complement Deficiency States and Infection: Epidemiology, Pathogenesis and Consequences of Neisserial and Other Infections in an Immune Deficiency. Medicine *63*, 243–273.
42. Nishizaka, H., Horiuchi, T., Zhu, Z.B., Fukumori, Y., Nagasawa, K., Hayashi, K., Krumdieck, R., Cobbs, C.G., Higuchi, M., Yasunaga, S., et al. (1996). Molecular bases for inherited human complement component C6 deficiency in two unrelated individuals. J. Immunol. *156*, 2309–2315.
43. Nagata, M., Hara, T., Aoki, T., Mizuno, Y., Akeda, H., Inaba, S., Tsumoto, K., and Ueda, K. (1989). Inherited deficiency of ninth component of complement: An increased risk of meningococcal meningitis. J. Pediatr. *114*, 260–264.
44. Nishizaki, M. (1992). [The association between deficiency of terminal complement components and the occurrence of meningococcal meningitis]. Fukuoka Igaku Zasshi *83*, 201–208.
45. Kira, R., Ihara, K., Watanabe, K., Kanemitsu, S., Ahmed, S.U., Gondo, K., Takeshita, K., and Hara, T. (1999). Molecular epidemiology of C9 deficiency heterozygotes with an Arg95Stop mutation of the C9 gene in Japan. J. Hum. Genet. *44*, 109–111.
46. Higasa, K., Miyake, N., Yoshimura, J., Okamura, K., Niihori, T., Saitsu, H., Doi, K., Shimizu, M., Nakabayashi, K., Aoki, Y., et al. (2016). Human genetic variation database, a reference database of genetic variations in the Japanese population. J. Hum. Genet. *61*, 547–553.
47. Dean, M., Carrington, M., Winkler, C., Huttley, G.A., Smith, M.W., Allikmets, R., Goedert, J.J., Buchbinder, S.P., Vittinghoff, E., Gomperts, E., et al. (1996). Genetic Restriction of HIV-1 Infection and Progression to AIDS by a Deletion Allele of the CKR5 Structural Gene. Science *273*, 1856–1862.
48. Liu, R., Paxton, W.A., Choe, S., Ceradini, D., Martin, S.R., Horuk, R., MacDonald, M.E., Stuhlmann, H., Koup, R.A., and Landau, N.R. (1996). Homozygous Defect in HIV-1 Coreceptor Accounts for Resistance of Some Multiply-Exposed Individuals to HIV-1 Infection. Cell *86*, 367–377.
49. Samson, M., Libert, F., Doranz, B.J., Rucker, J., Liesnard, C., Farber, C.M., Saragosti, S., Lapoumeroulie, C., Cognaux, J., Forceille, C., et al. (1996). Resistance to HIV-1 infection in Caucasian individuals bearing mutant alleles of the CCR-5 chemokine receptor gene. Nature *382*, 722–725.
50. Murphy, S., Trqup, G., Hayward, A., Devor, E., and Coons, T. (1980). GENE ENRICHMENT IN AN AMERICAN INDIAN POPULATION: AN EXCESS OF SEVERE COMBINED IMMUNODEFICIENCY DISEASE. Lancet *316*, 502–505.
51. Li, L., Moshous, D., Zhou, Y., Wang, J., Xie, G., Salido, E., Hu, D., de Villartay, J.P., and Cowan, M.J. (2002). A Founder Mutation in Artemis, an SNM1-Like Protein, Causes SCID in Athabascan-Speaking Native Americans1. J. Immunol. *168*, 6323–6329.
52. Gayden, T., Sepulveda, F.E., Khuong-Quang, D.A., Pratt, J., Valera, E.T., Garrigue, A., Kelso, S., Sicheri, F., Mikael, L.G., Hamel, N., et al. (2018). Germline HAVCR2 mutations altering TIM-3 characterize subcutaneous panniculitis-like T cell lymphomas with hemophagocytic lymphohistiocytic syndrome. Nat. Genet. *50*, 1650–1657.
53. Al Qureshah, F., Le Pen, J., de Weerd, N.A., Moncada-Velez, M., Materna, M., Lin, D.C., Milisavljevic, B., Vianna, F., Bizien, L., Lorenzo, L., et al. (2025). A common form of dominant human IFNAR1 deficiency impairs IFN-α and -ω but not IFN-β-dependent immunity. J. Exp. Med. *222*, e20241413.
54. Zhang, G., deWeerd, N.A., Stifter, S.A., Liu, L., Zhou, B., Wang, W., Zhou, Y., Ying, B., Hu, X., Matthews, A.Y., et al. (2018). A proline deletion in IFNAR1 impairs IFN-signaling and underlies increased resistance to tuberculosis in humans. Nat. Commun. *9*, 85.
55. Bastard, P., Hsiao, K.C., Zhang, Q., Choin, J., Best, E., Chen, J., Gervais, A., Bizien, L., Materna, M., Harmant, C., et al. (2022). A loss-of-function IFNAR1 allele in Polynesia underlies severe viral diseases in homozygotes. J. Exp. Med. *219*, e20220028.
56. Duncan, C.J.A., Skouboe, M.K., Howarth, S., Hollensen, A.K., Chen, R., Børresen, M.L., Thompson, B.J., Stremenova Spegarova, J., Hatton, C.F., Stæger, F.F., et al. (2022). Life-threatening viral disease in a novel form of autosomal recessive IFNAR2 deficiency in the Arctic. J. Exp. Med. *219*, e20212427.
57. Pannicke, U., Baumann, B., Fuchs, S., Henneke, P., Rensing-Ehl, A., Rizzi, M., Janda, A., Hese, K., Schlesier, M., Holzmann, K., Borte, S., et al. (2013). Deficiency of Innate and Acquired Immunity Caused by an IKBKB Mutation. N. Engl. J. Med. *369*, 2504–2514.
58. Rubin, T.S., Rockman-Greenberg, C., Van Caeseele, P., Cuvelier, G.D.E., Kwan, L., and Schroeder, M.L. (2018). Newborn Screening for IKBKB Deficiency in Manitoba, Using Genetic Mutation Analysis. J. Clin. Immunol. *38*, 742–744.

59. French FMF Consortium (1997). A candidate gene for familial Mediterranean fever. Nat. Genet. *17*, 25–31.

60. The International FMF Consortium (1997). Ancient Missense Mutations in a New Member of the *RoRet* Gene Family Are Likely to Cause Familial Mediterranean Fever. Cell *90*, 797–807.

61. Shohat, M., and Halpern, G.J. (2011). Familial Mediterranean fever—A review. Genet. Med. *13*, 487–498.

62. Stoffman, N., Magal, N., Shohat, T., Lotan, R., Koman, S., Oron, A., Danon, Y., Halpern, G.J., Lifshitz, Y., and Shohat, M. (2000). Higher than expected carrier rates for familial Mediterranean fever in various Jewish ethnic groups. Eur. J. Hum. Genet. *8*, 307–310.

63. Honda, Y., Maeda, Y., Izawa, K., Shiba, T., Tanaka, T., Nakaseko, H., Nishimura, K., Mukoyama, H., Isa-Nishitani, M., Miyamoto, T., et al. (2021). Rapid Flow Cytometry-Based Assay for the Functional Classification of MEFV Variants. J. Clin. Immunol. *41*, 1187–1197.

64. Ahmad, T., Armuzzi, A., Bunce, M., Mulcahy-Hawes, K., Marshall, S.E., Orchard, T.R., Crawshaw, J., Large, O., de Silva, A., Cook, J.T., et al. (2002). The molecular classification of the clinical manifestations of Crohn's disease. Gastroenterology *122*, 854–866.

65. Bonen, D.K., Ogura, Y., Nicolae, D.L., Inohara, N., Saab, L., Tanabe, T., Chen, F.F., Foster, S.J., Duerr, R.H., Brant, S.R., et al. (2003). Crohn's disease-associated NOD2 variants share a signaling defect in response to lipopolysaccharide and peptidoglycan. Gastroenterology *124*, 140–146.

66. Materna, M., Delmonte, O.M., Bosticardo, M., Momenilandi, M., Conrey, P.E., Charmeteau-De Muylder, B., Bravetti, C., Bellworthy, R., Cederholm, A., Staels, F., et al. (2024). The immunopathological landscape of human pre-TCRα deficiency: From rare to common variants. Science *383*, eadh4059.

67. Boisson-Dupuis, S., Ramirez-Alejo, N., Li, Z., Patin, E., Rao, G., Kerner, G., Lim, C.K., Krementsov, D.N., Hernandez, N., Ma, C.S., et al. (2018). Tuberculosis and impaired IL-23–dependent IFN-γ immunity in humans homozygous for a common TYK2 missense variant. Sci. Immunol. *3*, eaau8714.

68. Kerner, G., Ramirez-Alejo, N., Seeleuthner, Y., Yang, R., Ogishi, M., Cobat, A., Patin, E., Quintana-Murci, L., Boisson-Dupuis, S., Casanova, J.L., and Abel, L. (2019). Homozygosity for TYK2 P1104A underlies tuberculosis in about 1% of patients in a cohort of European ancestry. Proc. Natl. Acad. Sci. USA *116*, 10430–10434. https://doi.org/10.1073/pnas.1903561116.

69. Figueroa, J.E., and Densen, P. (1991). Infectious diseases associated with complement deficiencies. Clin. Microbiol. Rev. *4*, 359–395.

70. Ellwanger, J.H., Kulmann-Leal, B., Kaminski, V.d.L., Rodrigues, A.G., Bragatte, M.A.d.S., and Chies, J.A.B. (2020). Beyond HIV infection: Neglected and varied impacts of CCR5 and CCR5Δ32 on viral diseases. Virus Res. *286*, 198040.

71. Li, L., Drayna, D., Hu, D., Hayward, A., Gahagan, S., Pabst, H., and Cowan, M.J. (1998). The Gene for Severe Combined Immunodeficiency Disease in Athabascan-Speaking Native Americans Is Located on Chromosome 10p. Am. J. Hum. Genet. *62*, 136–144.

72. Kim, Y., Coomarasamy, C., and Jarrett, P. (2020). The epidemiology of subcutaneous panniculitis-like alpha-beta T-cell lymphoma in New Zealand. Australas. J. Dermatol. *61*, e196–e199.

73. Cuvelier, G.D.E., Rubin, T.S., Junker, A., Sinha, R., Rosenberg, A.M., Wall, D.A., and Schroeder, M.L. (2019). Clinical presentation, immunologic features, and hematopoietic stem cell transplant outcomes for IKBKB immune deficiency. Clin. Immunol. *205*, 138–147.

74. Ben-Chetrit, E. (2024). Old paradigms and new concepts in familial Mediterranean fever (FMF): an update 2023. Rheumatology *63*, 309–318.

75. Gershoni-Baruch, R., Brik, R., Shinawi, M., and Livneh, A. (2002). The differential contribution of MEFV mutant alleles to the clinical profile of familial Mediterranean fever. Eur. J. Hum. Genet. *10*, 145–149.

76. Cazeneuve, C., Sarkisian, T., Pêcheux, C., Dervichian, M., Nédelec, B., Reinert, P., Ayvazyan, A., Kouyoumdjian, J.C., Ajrapetyan, H., Delpech, M., et al. (1999). MEFV-Gene Analysis in Armenian Patients with Familial Mediterranean Fever: Diagnostic Value and Unfavorable Renal Prognosis of the M694V Homozygous Genotype—Genetic and Therapeutic Implications. Am. J. Hum. Genet. *65*, 88–97.

77. Medlej-Hashim, M., Rawashdeh, M., Chouery, E., Mansour, I., Delague, V., Lefranc, G., Naman, R., Loiselet, J., and Mégarbané, A. (2000). Genetic screening of fourteen mutations in Jordanian familial Mediterranean fever patients. Hum. Mutat. *15*, 384.

78. Jéru, I., Hentgen, V., Cochet, E., Duquesnoy, P., Le Borgne, G., Grimprel, E., Stojanovic, K.S., Karabina, S., Grateau, G., and Amselem, S. (2013). The Risk of Familial Mediterranean Fever in MEFV Heterozygotes: A Statistical Approach. PLoS One *8*, e68431.

79. Eyal, O., Shinar, Y., Pras, M., and Pras, E. (2020). Familial Mediterranean fever: Penetrance of the p.[Met694Val];[Glu148Gln] and p.[Met694Val];[=] genotypes. Hum. Mutat. *41*, 1866–1870.

80. Yazdanyar, S., Kamstrup, P.R., Tybjærg-Hansen, A., and Nordestgaard, B.G. (2010). Penetrance of NOD2/CARD15 genetic variants in the general population. CMAJ (Can. Med. Assoc. J.) *182*, 661–665.

81. Xie, C.B., Jane-Wit, D., and Pober, J.S. (2020). Complement Membrane Attack Complex. Am. J. Pathol. *190*, 1138–1150.

82. Lim, D., Gewurz, A., Lint, T.F., Ghaze, M., Sepheri, B., and Gewurz, H. (1976). Absence of the sixth component of complement in a patient with repeated episodes of meningococcal meningitis. J. Pediatr. *89*, 42–47.

83. Rosain, J., Ngo, S., Bordereau, P., Poulain, N., Roncelin, S., Vieira Martins, P., Dragon-Durey, M.A., and Frémeaux-Bacchi, V. (2014). Complement deficiencies and human diseases. Ann. Biol. Clin. *72*, 271–280.

84. Rosain, J., Hong, E., Fieschi, C., Martins, P.V., El Sissy, C., Deghmane, A.E., Ouachée, M., Thomas, C., Launay, D., de Pontual, L., et al. (2017). Strains Responsible for Invasive Meningococcal Disease in Patients With Terminal Complement Pathway Deficiencies. J. Infect. Dis. *215*, 1331–1338.

85. Raoult, D., Gallais, H., Casanova, P., and Lesavre, P. (1987). Haemophilus parainfluenzae Meningitis in an Adult With an Inherited Deficiency of the Seventh Component of Complement. Arch. Intern. Med. *147*, 2214.

86. Rameix-Welti, M.-A., Régnier, C.H., Bienaimé, F., Blouin, J., Schifferli, J., Fridman, W.H., Sautès-Fridman, C., and Frémeaux-Bacchi, V. (2007). Hereditary complement C7 deficiency in nine families: Subtotal C7 deficiency revisited. Eur. J. Immunol. *37*, 1377–1385.

87. Leddy, J.P., Frank, M.M., Gaither, T., Baum, J., and Klemperer, M.R. (1974). Hereditary deficiency of the sixth component of complement in man. I. Immunochemical, biologic, and family studies. J. Clin. Investig. *53*, 544–553.

88. Hobart, M.J., Fernie, B.A., Fijen, K.A., and Orren, A. (1998). The molecular basis of C6 deficiency in the western Cape, South Africa. Hum. Genet. *103*, 506–512.

89. Zhu, Z.B., Totemchokchyakarn, K., Atkinson, T.P., and Volanakis, J.E. (1998). Molecular defects leading to human complement component C6 deficiency in an African-American family. Clin. Exp. Immunol. *111*, 91–96.

90. Parikh, S.R., Campbell, H., Bettinger, J.A., Harrison, L.H., Marshall, H.S., Martinon-Torres, F., Safadi, M.A., Shao, Z., Zhu, B., von Gottberg, A., et al. (2020). The everchanging epidemiology of meningococcal disease worldwide and the potential for prevention through vaccination. J. Infect. *81*, 483–498.

91. Stephens, D.S., Greenwood, B., and Brandtzaeg, P. (2007). Epidemic meningitis, meningococcaemia, and *Neisseria meningitidis*. Lancet *369*, 2196–2210.

92. Inai, S., Kitamura, H., Hiramatsu, S., and Nagaki, K. (1979). Deficiency of the ninth component of complement in man. J. Clin. Lab. Immunol. *2*, 85–87.

93. Inaba, S., Okochi, K., Fukada, K., Kinoshita, S., Maeda, Y., and Yoshinari, M. (1987). The occurrence of precipitating antibodies in transfused Japanese patients with hereditary ninth component of complement deficiency and frequency of C9 deficiency. Transfusion *27*, 475–477.

94. Kira, R., Ihara, K., Takada, H., Gondo, K., and Hara, T. (1998). Nonsense mutation in exon 4 of human complement C9 gene is the major cause of Japanese complement C9 deficiency. Hum. Genet. *102*, 605–610.

95. Würzner, R. (2003). Deficiencies of the complement MAC II gene cluster (C6, C7, C9): is subtotal C6 deficiency of particular evolutionary benefit? Clin. Exp. Immunol. *133*, 156–159.

96. Janeway, T.C. (1908). AN UNUSUAL PAROXYSMAL SYNDROME, PROBABLY ALLIED TO RECURRENT VOMITING, WITH A STUDY OF THE NITROGEN METABOLISM. Arch. Intern. Med. *II*, 214.

97. SIEGAL, S. (1945). Benign paroxysmal peritonitis. Ann. Intern. Med. *23*, 1–21.

98. Siegal, S. (1949). Benign Paroxysmal Peritonitis—Second Series. Gastroenterology *12*, 234–247.

99. Balow, J.E., Shelton, D.A., Orsborn, A., Mangelsdorf, M., Aksentijevich, I., Blake, T., Sood, R., Gardner, D., Liu, R., Pras, E., et al. (1997). A High-Resolution Genetic Map of the Familial Mediterranean Fever Candidate Region Allows Identification of Haplotype-Sharing among Ethnic Groups. Genomics *44*, 280–291.

100. Xu, H., Yang, J., Gao, W., Li, L., Li, P., Zhang, L., Gong, Y.N., Peng, X., Xi, J.J., Chen, S., et al. (2014). Innate immune sensing of bacterial modifications of Rho GTPases by the Pyrin inflammasome. Nature *513*, 237–241.

101. Van Gorp, H., Saavedra, P.H.V., de Vasconcelos, N.M., Van Opdenbosch, N., Vande Walle, L., Matusiak, M., Prencipe, G., Insalaco, A., Van Hauwermeiren, F., Demon, D., et al. (2016). Familial Mediterranean fever mutations lift the obligatory requirement for microtubules in Pyrin inflammasome activation. Proc. Natl. Acad. Sci. USA *113*, 14384–14389.

102. Park, Y.H., Wood, G., Kastner, D.L., and Chae, J.J. (2016). Pyrin inflammasome activation and RhoA signaling in the autoinflammatory diseases FMF and HIDS. Nat. Immunol. *17*, 914–921.

103. Rogers, D.B., Shohat, M., Petersen, G.M., Bickal, J., Congleton, J., Schwabe, A.D., and Rotter, J.I. (1989). Familial Mediterranean fever in Armenians: Autosomal recessive inheritance with high gene frequency. Am. J. Med. Genet. *34*, 168–172.

104. Daniels, M., Shohat, T., Brenner-Ullman, A., and Shohat, M. (1995). Familial Mediterranean fever: High gene frequency among the non-Ashkenazic and ashkenazic Jewish populations in Israel. Am. J. Med. Genet. *55*, 311–314.

105. Yuval, Y., Hemo-Zisser, M., Zemer, D., Sohar, E., and Pras, M. (1995). Dominant inheritance in two families with familial Mediterranean fever (FMF). Am. J. Med. Genet. *57*, 455–457.

106. Li, Z., Akar, S., Yarkan, H., Lee, S.K., Çetin, P., Can, G., Kenar, G., Çapa, F., Pamuk, O.N., Pehlivan, Y., et al. (2019). Genome-wide association study in Turkish and Iranian populations identify rare familial Mediterranean fever gene (MEFV) polymorphisms associated with ankylosing spondylitis. PLoS Genet. *15*, e1008038.

107. Chung, L.K., Park, Y.H., Zheng, Y., Brodsky, I.E., Hearing, P., Kastner, D.L., Chae, J.J., and Bliska, J.B. (2016). The *Yersinia* Virulence Factor YopM Hijacks Host Kinases to Inhibit Type III Effector-Triggered Activation of the Pyrin Inflammasome. Cell Host Microbe *20*, 296–306.

108. Patin, E. (2020). Plague as a cause for familial Mediterranean fever. Nat. Immunol. *21*, 833–834.

109. Park, Y.H., Remmers, E.F., Lee, W., Ombrello, A.K., Chung, L.K., Shilei, Z., Stone, D.L., Ivanov, M.I., Loeven, N.A., Barron, K.S., et al. (2020). Ancient familial Mediterranean fever mutations in human pyrin and resistance to Yersinia pestis. Nat. Immunol. *21*, 857–867.

110. Yui, M.A., and Rothenberg, E.V. (2014). Developmental gene networks: a triathlon on the course to T cell identity. Nat. Rev. Immunol. *14*, 529–545.

111. Fischer, A., Notarangelo, L.D., Neven, B., Cavazzana, M., and Puck, J.M. (2015). Severe combined immunodeficiencies and related disorders. Nat. Rev. Dis. Primers *1*, 15061.

112. Jones, J.F., Ritenbaugh, C.K., Spence, M.A., and Hayward, A. (1991). Severe combined immunodeficiency among the Navajo. I. Characterization of phenotypes, epidemiology, and population genetics. Hum. Biol. *63*, 669–682.

113. Moshous, D., Callebaut, I., de Chasseval, R., Corneo, B., Cavazzana-Calvo, M., Le Deist, F., Tezcan, I., Sanal, O., Bertrand, Y., Philippe, N., et al. (2001). Artemis, a Novel DNA Double-Strand Break Repair/V(D)J Recombination Protein, Is Mutated in Human Severe Combined Immune Deficiency. Cell *105*, 177–186.

114. Santisteban, I., Arredondo-Vega, F.X., Kelly, S., Loubser, M., Meydan, N., Roifman, C., Howell, P.L., Bowen, T., Weinberg, K.I., and Schroeder, M.L. (1995). Three new adenosine deaminase mutations that define a splicing enhancer and cause severe and partial phenotypes: implications for evolution of a CpG hotspot and expression of a transduced ADA cDNA. Hum. Mol. Genet. *4*, 2081–2087.

115. Zhang, Q., Lenardo, M.J., and Baltimore, D. (2017). 30 Years of NF-κB: A Blossoming of Relevance to Human Pathobiology. Cell *168*, 37–57.

116. Erickson, R.P. (2021). Autosomal recessive diseases among the Athabaskans of the southwestern United States: anthropological, medical, and scientific aspects. J. Appl. Genet. *62*, 445–453.

117. DeGiorgio, M., Jakobsson, M., and Rosenberg, N.A. (2009). Out of Africa: modern human origins special feature: explaining worldwide patterns of human genetic variation using a coalescent-based serial founder model of migration outward from Africa. Proc. Natl. Acad. Sci. USA *106*, 16057–16062.

118. Furin, J., Cox, H., and Pai, M. (2019). Tuberculosis. Lancet *393*, 1642–1656.

119. Mimouni, J. (1951). Notre expérience de trois années de vaccination à Constantine; étude de 25 cas de complications. Alger. Med. *55*, 1138–1147.

120. Bustamante, J. (2020). Mendelian susceptibility to mycobacterial disease: recent discoveries. Hum. Genet. *139*, 993–1000.

121. Ogishi, M., Yang, R., Rosain, J., Bustamante, J., Casanova, J.L., and Boisson-Dupuis, S. (2023). Inborn errors of human transcription factors governing IFN-γ antimycobacterial immunity. Curr. Opin. Immunol. *81*, 102296.

122. Le Voyer, T., Neehus, A.L., Yang, R., Ogishi, M., Rosain, J., Alroqi, F., Alshalan, M., Blumental, S., Al Ali, F., Khan, T., et al. (2021). Inherited deficiency of stress granule ZNFX1 in patients with monocytosis and mycobacterial disease. Proc. Natl. Acad. Sci. USA *118*, e2102804118.

123. Martin-Fernandez, M., Buta, S., Le Voyer, T., Li, Z., Dynesen, L.T., Vuillier, F., Franklin, L., Ailal, F., Muglia Amancio, A., Malle, L., et al. (2022). A partial form of inherited human USP18 deficiency underlies infection and inflammation. J. Exp. Med. *219*, e20211273.

124. Rosain, J., Neehus, A.L., Manry, J., Yang, R., Le Pen, J., Daher, W., Liu, Z., Chan, Y.H., Tahuil, N., Türel, Ö., et al. (2023). Human IRF1 governs macrophagic IFN-γ immunity to mycobacteria. Cell *186*, 621–645.e33.

125. Bohlen, J., Zhou, Q., Philippot, Q., Ogishi, M., Rinchai, D., Nieminen, T., Seyedpour, S., Parvaneh, N., Rezaei, N., Yazdanpanah, N., et al. (2023). Human MCTS1-dependent translation of JAK2 is essential for IFN-γ immunity to mycobacteria. Cell *186*, 5114–5134.e27.

126. Neehus, A.-L., Carey, B., Landekic, M., Panikulam, P., Deutsch, G., Ogishi, M., Arango-Franco, C.A., Philippot, Q., Modaresi, M., Mohammadzadeh, I., et al. (2024). Human inherited CCR2 deficiency underlies progressive polycystic lung disease. Cell *187*, 3460.

127. Boisson-Dupuis, S. (2020). The monogenic basis of human tuberculosis. Hum. Genet. *139*, 1001–1009.

128. Altare, F., Ensser, A., Breiman, A., Reichenbach, J., Baghdadi, J.E., Fischer, A., Emile, J.F., Gaillard, J.L., Meinl, E., and Casanova, J.L. (2001). Interleukin-12 receptor beta1 deficiency in a patient with abdominal tuberculosis. J. Infect. Dis. *184*, 231–236.

129. Kreins, A.Y., Ciancanelli, M.J., Okada, S., Kong, X.F., Ramírez-Alejo, N., Kilic, S.S., El Baghdadi, J., Nonoyama, S., Mahdaviani, S.A., Ailal, F., et al. (2015). Human TYK2 deficiency: Mycobacterial and viral infections without hyper-IgE syndrome. J. Exp. Med. *212*, 1641–1662.

130. Ogishi, M., Arias, A.A., Yang, R., Han, J.E., Zhang, P., Rinchai, D., Halpern, J., Mulwa, J., Keating, N., Chrabieh, M., et al. (2022). Impaired IL-23–dependent induction of IFN-γ underlies mycobacterial disease in patients with inherited TYK2 deficiency. J. Exp. Med. *219*, e20220094.

131. Martínez-Barricarte, R., Markle, J.G., Ma, C.S., Deenick, E.K., Ramírez-Alejo, N., Mele, F., Latorre, D., Mahdaviani, S.A., Aytekin, C., Mansouri, D., Bryant, V.L., et al. (2018). Human IFN-γ immunity to mycobacteria is governed by both IL-12 and IL-23. Sci. Immunol. *3*.

132. Bycroft, C., Freeman, C., Petkova, D., Band, G., Elliott, L.T., Sharp, K., Motyer, A., Vukcevic, D., Delaneau, O., O'Connell, J., et al. (2018). The UK Biobank resource with deep phenotyping and genomic data. Nature *562*, 203–209.

133. Diogo, D., Bastarache, L., Liao, K.P., Graham, R.R., Fulton, R.S., Greenberg, J.D., Eyre, S., Bowes, J., Cui, J., Lee, A., et al. (2015). TYK2 Protein-Coding Variants Protect against Rheumatoid Arthritis and Autoimmunity, with No Evidence of Major Pleiotropic Effects on Non-Autoimmune Complex Traits. PLoS One *10*, e0122271.

134. Kerner, G., Laval, G., Patin, E., Boisson-Dupuis, S., Abel, L., Casanova, J.L., and Quintana-Murci, L. (2021). Human ancient DNA analyses reveal the high burden of tuberculosis in Europeans over the last 2,000 years. Am. J. Hum. Genet. *108*, 517–524.

135. Paulson, T. (2013). Epidemiology: A mortal foe. Nature *502*, S2–S3.

136. Akbari, A., Barton, A.R., Gazal, S., Li, Z., Kariminejad, M., Perry, A., Zeng, Y., Mittnik, A., Patterson, N., Mah, M., et al. (2024). Pervasive findings of directional selection realize the promise of ancient DNA to elucidate human adaptation. Preprint at bioRxiv. https://doi.org/10.1101/2024.09.14.613021.

137. Hoffmann, H.-H., Schneider, W.M., and Rice, C.M. (2015). Interferons and viruses: an evolutionary arms race of molecular interactions. Trends Immunol. *36*, 124–138.

138. Isaacs, A., Lindenmann, J., and Andrewes, C.H. (1997). Virus interference. I. The interferon. Proc. Roy. Soc. Lond. B Biol. Sci. *147*, 258–267.

139. Ciancanelli, M.J., Huang, S.X.L., Luthra, P., Garner, H., Itan, Y., Volpi, S., Lafaille, F.G., Trouillet, C., Schmolke, M., Albrecht, R.A., et al. (2015). Life-threatening influenza and impaired interferon amplification in human IRF7 deficiency. Science *348*, 448–453.

140. Duncan, C.J.A., Mohamad, S.M.B., Young, D.F., Skelton, A.J., Leahy, T.R., Munday, D.C., Butler, K.M., Morfopoulou, S., Brown, J.R., Hubank, M., et al. (2015). Human IFNAR2 deficiency: Lessons for antiviral immunity. Sci. Transl. Med. *7*, 307ra154.

141. Hernandez, N., Melki, I., Jing, H., Habib, T., Huang, S.S.Y., Danielson, J., Kula, T., Drutman, S., Belkaya, S., Rattina, V., et al. (2018). Life-threatening influenza pneumonitis in a child with inherited IRF9 deficiency. J. Exp. Med. *215*, 2567–2585.

142. Hernandez, N., Bucciol, G., Moens, L., Le Pen, J., Shahrooei, M., Goudouris, E., Shirkani, A., Changi-Ashtiani, M., Rokni-Zadeh, H., Sayar, E.H., et al. (2019). Inherited IFNAR1 deficiency in otherwise healthy patients with adverse reaction to measles and yellow fever live vaccines. J. Exp. Med. *216*, 2057–2070.

143. Zhang, Q., Bastard, P., Liu, Z., Le Pen, J., Moncada-Velez, M., Chen, J., Ogishi, M., Sabli, I.K.D., Hodeib, S., Korol, C., et al. (2020). Inborn errors of type I IFN immunity in patients with life-threatening COVID-19. Science *370*, eabd4570.

144. Bastard, P., Manry, J., Chen, J., Rosain, J., Seeleuthner, Y., AbuZaitun, O., Lorenzo, L., Khan, T., Hasek, M., Hernandez, N., Bigio, B., et al. (2021). Herpes simplex encephalitis in a patient with a distinctive form of inherited IFNAR1 deficiency. J. Clin. Investig. *131*.

145. Zhang, Q., Matuozzo, D., Le Pen, J., Lee, D., Moens, L., Asano, T., Bohlen, J., Liu, Z., Moncada-Velez, M., Kendir-Demirkol, Y., et al. (2022). Recessive inborn errors of type I IFN immunity in children with COVID-19 pneumonia. J. Exp. Med. *219*, e20220131.

146. Campbell, T.M., Liu, Z., Zhang, Q., Moncada-Velez, M., Covill, L.E., Zhang, P., Alavi Darazam, I., Bastard, P., Bizien, L., Bucciol, G., et al. (2022). Respiratory viral infections in otherwise healthy humans with inherited IRF7 deficiency. J. Exp. Med. *219*, e20220202.

147. Bucciol, G., Moens, L., Ogishi, M., Rinchai, D., Matuozzo, D., Momenilandi, M., Kerrouche, N., Cale, C.M., Treffeisen, E.R., Al Salamah, M., et al. (2023). Human inherited complete STAT2 deficiency underlies inflammatory viral diseases. J. Clin. Investig. *133*, e168321.

148. Abolhassani, H., Landegren, N., Bastard, P., Materna, M., Modaresi, M., Du, L., Aranda-Guillén, M., Sardh, F., Zuo, F., Zhang, P., et al. (2022). Inherited IFNAR1 Deficiency in a Child with Both Critical COVID-19 Pneumonia and Multisystem Inflammatory Syndrome. J. Clin. Immunol. *42*, 471–483. https://doi.org/10.1007/s10875-022-01215-7.

149. Gothe, F., Hatton, C.F., Truong, L., Klimova, Z., Kanderova, V., Fejtkova, M., Grainger, A., Bigley, V., Perthen, J., Mitra, D., et al. (2022). A Novel Case of Homozygous Interferon Alpha/Beta Receptor Alpha Chain (IFNAR1) Deficiency With Hemophagocytic Lymphohistiocytosis. Clin. Infect. Dis. *74*, 136–139.

150. Khanmohammadi, S., Rezaei, N., Khazaei, M., and Shirkani, A. (2022). A Case of Autosomal Recessive Interferon Alpha/Beta Receptor Alpha Chain (IFNAR1) Deficiency with Severe COVID-19. J. Clin. Immunol. *42*, 19–24.

151. Passarelli, C., Civino, A., Rossi, M.N., Cifaldi, L., Lanari, V., Moneta, G.M., Caiello, I., Bracaglia, C., Montinaro, R., Novelli, A., et al. (2020). IFNAR2 Deficiency Causing Dysregulation of NK Cell Functions and Presenting With Hemophagocytic Lymphohistiocytosis. Front. Genet. *11*, 937.

152. Bastard, P., Michailidis, E., Hoffmann, H.H., Chbihi, M., Le Voyer, T., Rosain, J., Philippot, Q., Seeleuthner, Y., Gervais, A., Materna, M., et al. (2021). Auto-antibodies to type I IFNs can underlie adverse reactions to yellow fever live attenuated vaccine. J. Exp. Med. *218*, e20202486.

153. Lamborn, I.T., Jing, H., Zhang, Y., Drutman, S.B., Abbott, J.K., Munir, S., Bade, S., Murdock, H.M., Santos, C.P., Brock, L.G., et al. (2017). Recurrent rhinovirus infections in a child with inherited MDA5 deficiency. J. Exp. Med. *214*, 1949–1972.

154. Asgari, S., Schlapbach, L.J., Anchisi, S., Hammer, C., Bartha, I., Junier, T., Mottet-Osman, G., Posfay-Barbe, K.M., Longchamp, D., Stocker, M., et al. (2017). Severe viral respiratory infections in children with IFIH1 loss-of-function mutations. Proc. Natl. Acad. Sci. USA *114*, 8342–8347.

155. Lim, H.K., Huang, S.X.L., Chen, J., Kerner, G., Gilliaux, O., Bastard, P., Dobbs, K., Hernandez, N., Goudin, N., Hasek, M.L., et al. (2019). Severe influenza pneumonitis in children with inherited TLR3 deficiency. J. Exp. Med. *216*, 2038–2056.

156. Le Voyer, T., Sakata, S., Tsumura, M., Khan, T., Esteve-Sole, A., Al-Saud, B.K., Gungor, H.E., Taur, P., Jeanne-Julien, V., Christiansen, M., et al. (2021). Genetic, Immunological, and Clinical Features of 32 Patients with Autosomal Recessive STAT1 Deficiency. J. Immunol. *207*, 133–152.

157. Bastard, P., Rosen, L.B., Zhang, Q., Michailidis, E., Hoffmann, H.H., Zhang, Y., Dorgham, K., Philippot, Q., Rosain, J., Béziat, V., Manry, J., et al. (2020). Autoantibodies against type I IFNs in patients with life-threatening COVID-19. Science *370*, eabd4585.

158. Gervais, A., Rovida, F., Avanzini, M.A., Croce, S., Marchal, A., Lin, S.C., Ferrari, A., Thorball, C.W., Constant, O., Le Voyer, T., et al. (2023). Auto-antibodies neutralizing type I IFNs underlie West Nile virus encephalitis in ∼40% of patients. J. Exp. Med. *220*, e20230661.

CellPress
OPEN ACCESS

159. Zhang, Q., Pizzorno, A., Miorin, L., Bastard, P., Gervais, A., Le Voyer, T., Bizien, L., Manry, J., Rosain, J., Philippot, Q., et al. (2022). Autoantibodies against type I IFNs in patients with critical influenza pneumonia. J. Exp. Med. *219*, e20220514.

160. Meyts, I. (2022). Null IFNAR1 and IFNAR2 alleles are surprisingly common in the Pacific and Arctic. J. Exp. Med. *219*, e20220491.

161. Quintana-Murci, L. (2019). Human Immunology through the Lens of Evolutionary Genetics. Cell *177*, 184–199.

162. Mathieson, I., Lazaridis, I., Rohland, N., Mallick, S., Patterson, N., Roodenberg, S.A., Harney, E., Stewardson, K., Fernandes, D., Novak, M., et al. (2015). Genome-wide patterns of selection in 230 ancient Eurasians. Nature *528*, 499–503.

163. Barrie, W., Yang, Y., Irving-Pease, E.K., Attfield, K.E., Scorrano, G., Jensen, L.T., Armen, A.P., Dimopoulos, E.A., Stern, A., Refoyo-Martinez, A., et al. (2024). Elevated genetic risk for multiple sclerosis emerged in steppe pastoralist populations. Nature *625*, 321–328.

164. Kerner, G., Neehus, A.L., Philippot, Q., Bohlen, J., Rinchai, D., Kerrouche, N., Puel, A., Zhang, S.Y., Boisson-Dupuis, S., Abel, L., et al. (2023). Genetic adaptation to pathogens and increased risk of inflammatory disorders in post-Neolithic Europe. Cell Genom. *3*, 100248.

165. Migueles, S.A., Sabbaghian, M.S., Shupert, W.L., Bettinotti, M.P., Marincola, F.M., Martino, L., Hallahan, C.W., Selig, S.M., Schwartz, D., Sullivan, J., and Connors, M. (2000). HLA B*5701 is highly associated with restriction of virus replication in a subgroup of HIV-infected long term nonprogressors. Proc. Natl. Acad. Sci. USA *97*, 2709–2714.

166. International HIV Controllers Study; Pereyra, F., Jia, X., McLaren, P.J., Telenti, A., de Bakker, P.I.W., Walker, B.D., Ripke, S., Brumme, C.J., Pulit, S.L., and et al.. (2010). The Major Genetic Determinants of HIV-1 Control Affect HLA Class I Peptide Presentation. Science *330*, 1551–1557.

167. Brewerton, D.A., Hart, F.D., Nicholls, A., Caffrey, M., James, D.C., and Sturrock, R.D. (1973). Ankylosing spondylitis and HL-A 27. Lancet *1*, 904–907.

168. International Genetics of Ankylosing Spondylitis Consortium IGAS; Cortes, A., Hadler, J., Pointon, J.P., Robinson, P.C., Karaderi, T., Leo, P., Cremin, K., Pryce, K., Harris, J., and et al.. (2013). Identification of multiple risk variants for ankylosing spondylitis through high-density genotyping of immune-related loci. Nat. Genet. *45*, 730–738.

169. Brewerton, D.A., Caffrey, M., Nicholls, A., Walters, D., Oates, J.K., and James, D.C. (1973). REITER'S DISEASE AND HL-A 27. Lancet *302*, 996–998.

170. McMichael, A., and Bowness, P. (2002). HLA-B27: natural function and pathogenic role in spondyloarthritis. Arthritis Res. *4*, S153–S158.

171. Van Linden, S.M.D., Valkenburg, H.A., Jongh, B.M.D., and Cats, A. (1984). The risk of developing ankylosing spondylitis in HLA-B27 positive individuals. Arthritis Rheum. *27*, 241–249.

172. Christy, M., Green, A., Christau, B., Kromann, H., Nerup, J., Platz, P., Thomsen, M., Ryder, L.P., and Svejgaard, A. (1979). Studies of the HLA System and Insulin-dependent Diabetes Mellitus. Diabetes Care *2*, 209–214.

173. Rotter, J.I., Anderson, C.E., Rubin, R., Congleton, J.E., Terasaki, P.I., and Rimoin, D.L. (1983). HLA Genotypic Study of Insulin-dependent Diabetes: The Excess of DR3/DR4 Heterozygotes Allows Rejection of the Recessive Hypothesis. Diabetes *32*, 169–174.

174. Aly, T.A., Ide, A., Jahromi, M.M., Barker, J.M., Fernando, M.S., Babu, S.R., Yu, L., Miao, D., Erlich, H.A., Fain, P.R., et al. (2006). Extreme genetic risk for type 1A diabetes. Proc. Natl. Acad. Sci. USA *103*, 14074–14079.

175. Erlich, H., Valdes, A.M., Noble, J., Carlson, J.A., Varney, M., Concannon, P., Mychaleckyj, J.C., Todd, J.A., Bonella, P., Fear, A.L., et al. (2008). HLA DR-DQ Haplotypes and Genotypes and Type 1 Diabetes Risk: Analysis of the Type 1 Diabetes Genetics Consortium Families. Diabetes *57*, 1084–1092.

176. Emery, L.M., Babu, S., Bugawan, T.L., Norris, J.M., Erlich, H.A., Eisenbarth, G.S., and Rewers, M. (2005). Newborn HLA-DR,DQ genotype screening: age- and ethnicity-specific type 1 diabetes risk estimates. Pediatr. Diabetes *6*, 136–144.

177. Blackwell, J.M., Jamieson, S.E., and Burgner, D. (2009). HLA and Infectious Diseases. Clin. Microbiol. Rev. *22*, 370–385.

178. Trowsdale, J., and Knight, J.C. (2013). Major histocompatibility complex genomics and human disease. Annu. Rev. Genomics Hum. Genet. *14*, 301–323.

179. Dendrou, C.A., Petersen, J., Rossjohn, J., and Fugger, L. (2018). HLA variation and disease. Nat. Rev. Immunol. *18*, 325–339.

180. Roda, G., Chien, Ng S., Kotze, P.G., Argollo, M., Panaccione, R., Spinelli, A., Kaser, A., Peyrin-Biroulet, L., and Danese, S. (2020). Crohn's disease. Nat. Rev. Dis. Primers *6*, 1–19.

181. Uhlig, H.H., and Schwerd, T. (2016). From Genes to Mechanisms: The Expanding Spectrum of Monogenic Disorders Associated with Inflammatory Bowel Disease. Inflamm. Bowel Dis. *22*, 202–212.

182. Sidiq, T., Yoshihama, S., Downs, I., and Kobayashi, K.S. (2016). Nod2: A Critical Regulator of Ileal Microbiota and Crohn's Disease. Front. Immunol. *7*, 367.

183. Kim, Y.-G., Shaw, M.H., Warner, N., Park, J.H., Chen, F., Ogura, Y., and Núñez, G. (2011). Crohn's Disease-Associated Nod2 Mutation Limits Production of Pro-Inflammatory Cytokines to Protect the Host from Enterococcus faecalis-Induced Lethality. J. Immunol. *187*, 2849–2852.

184. Deeks, S.G., Overbaugh, J., Phillips, A., and Buchbinder, S. (2015). HIV infection. Nat. Rev. Dis. Primers *1*, 15035.

185. Quillent, C., Oberlin, E., Braun, J., Rousset, D., Gonzalez-Canali, G., Métais, P., Montagnier, L., Virelizier, J.L., Arenzana-Seisdedos, F., and Beretta, A. (1998). HIV-1-resistance phenotype conferred by combination of two separate inherited mutations of *CCR5* gene. Lancet *351*, 14–18.

186. Ravn, K., Cobuccio, L., Muktupavela, R.A., Meisner, J., Benros, M.E., Korneliussen, T.S., Sikora, M., Willerslev, E., Allentoft, M.E., Irving-Pease, E.K., et al. (2023). Tracing the evolutionary path of the CCR5delta32 deletion via ancient and modern genomes. Preprint at medRxiv. https://doi.org/10.1101/2023.06.15.23290026.

187. Stephens, J.C., Reich, D.E., Goldstein, D.B., Shin, H.D., Smith, M.W., Carrington, M., Winkler, C., Huttley, G.A., Allikmets, R., Schriml, L., et al. (1998). Dating the Origin of the *CCR5-Δ32* AIDS-Resistance Allele by the Coalescence of Haplotypes. Am. J. Hum. Genet. *62*, 1507–1515.

188. Schliekelman, P., Garner, C., and Slatkin, M. (2001). Natural selection and resistance to HIV. Nature *411*, 545–546.

189. Galvani, A.P., and Slatkin, M. (2003). Evaluating plague and smallpox as historical selective pressures for the CCR5-Δ32 HIV-resistance allele. Proc. Natl. Acad. Sci. USA *100*, 15276–15279.

190. Glass, W.G., Lim, J.K., Cholera, R., Pletnev, A.G., Gao, J.L., and Murphy, P.M. (2005). Chemokine receptor CCR5 promotes leukocyte trafficking to the brain and survival in West Nile virus infection. J. Exp. Med. *202*, 1087–1098.

191. Willemze, R., Jansen, P.M., Cerroni, L., Berti, E., Santucci, M., Assaf, C., Canninga-van Dijk, M.R., Carlotti, A., Geerts, M.L., Hahtola, S., et al. (2008). Subcutaneous panniculitis-like T-cell lymphoma: definition, classification, and prognostic factors: an EORTC Cutaneous Lymphoma Group Study of 83 cases. Blood *111*, 838–845.

192. Swerdlow, S.H., Campo, E., Pileri, S.A., Harris, N.L., Stein, H., Siebert, R., Advani, R., Ghielmini, M., Salles, G.A., Zelenetz, A.D., and Jaffe, E.S. (2016). The 2016 revision of the World Health Organization classification of lymphoid neoplasms. Blood *127*, 2375–2390.

193. Gonzalez, C.L., Medeiros, L.J., Braziel, R.M., and Jaffe, E.S. (1991). T-Cell Lymphoma Involving Subcutaneous Tissue: A Clinicopathologic

Entity Commonly Associated with Hemophagocytic Syndrome. Am. J. Surg. Pathol. *15*, 17–27.

194. Pincus, L.B., LeBoit, P.E., McCalmont, T.H., Ricci, R., Buzio, C., Fox, L.P., Oliver, F., and Cerroni, L. (2009). Subcutaneous Panniculitis-Like T-Cell Lymphoma With Overlapping Clinicopathologic Features of Lupus Erythematosus: Coexistence of 2 Entities? Am. J. Dermatopathol. *31*, 520–526.

195. Joller, N., and Kuchroo, V.K. (2017). Tim-3, Lag-3, and TIGIT. Curr. Top. Microbiol. Immunol. *410*, 127–156.

196. Das, M., Zhu, C., and Kuchroo, V.K. (2017). Tim-3 and its role in regulating anti-tumor immunity. Immunol. Rev. *276*, 97–111.

197. Sonigo, G., Battistella, M., Beylot-Barry, M., Ingen-Housz-Oro, S., Franck, N., Barete, S., Boulinguez, S., Dereure, O., Bonnet, N., Socié, G., et al. (2020). HAVCR2 mutations are associated with severe hemophagocytic syndrome in subcutaneous panniculitis-like T-cell lymphoma. Blood *135*, 1058–1061.

198. Polprasert, C., Takeuchi, Y., Kakiuchi, N., Yoshida, K., Assanasen, T., Sitthi, W., Bunworasate, U., Pirunsarn, A., Wudhikarn, K., Lawasut, P., et al. (2019). Frequent germline mutations of HAVCR2 in sporadic subcutaneous panniculitis-like T-cell lymphoma. Blood Adv. *3*, 588–595.

199. Okamura, Y., Makishima, K., Suehara, Y., Suma, S., Abe, Y., Matsuoka, R., Sakamoto, T., Hattori, K., Yokoyama, Y., Kato, T., et al. (2024). Genetic profiles and clinical features in subcutaneous panniculitis-like T-cell lymphomas. Cancer Sci. *115*, 3788–3794.

200. Ishikawa, E., Miyake, Y., Hara, H., Saito, T., and Yamasaki, S. (2010). Germ-line elimination of electric charge on pre–T-cell receptor (TCR) impairs autonomous signaling for β-selection and TCR repertoire formation. Proc. Natl. Acad. Sci. USA *107*, 19979–19984.

201. Giblett, E.R., Anderson, J.E., Cohen, F., Pollara, B., and Meuwissen, H.J. (1972). ADENOSINE-DEAMINASE DEFICIENCY IN TWO PATIENTS WITH SEVERELY IMPAIRED CELLULAR IMMUNITY. Lancet *2*, 1067–1069.

202. Valerio, D., Dekker, B.M., Duyvesteyn, M.G., van der Voorn, L., Berkvens, T.M., van Ormondt, H., and van der Eb, A.J. (1986). One adenosine deaminase allele in a patient with severe combined immunodeficiency contains a point mutation abolishing enzyme activity. EMBO J. *5*, 113–119.

203. Minegishi, Y., Saito, M., Morio, T., Watanabe, K., Agematsu, K., Tsuchiya, S., Takada, H., Hara, T., Kawamura, N., Ariga, T., et al. (2006). Human Tyrosine Kinase 2 Deficiency Reveals Its Requisite Roles in Multiple Cytokine Signals Involved in Innate and Acquired Immunity. Immunity *25*, 745–755.

Cell Genomics, Volume *6*

Supplemental information

Monogenic disorders of immunity:

Common variants are not so rare

Vivien Béziat and Jean-Laurent Casanova

Monogenic disorders of immunity: common variants are not so rare

Vivien Béziat and Jean-Laurent Casanova

Summary

Initial submission:	Received : Jan 14, 2025
	Scientific editor: Sara Rohban
First round of review:	Number of reviewers: 3<br>Revision invited : Apr 16, 2025<br>Revision received : Aug 25, 2025
Second round of review:	Number of reviewers: 2<br>Revision invited : Oct 07, 2025<br>Revision received : Oct 17, 2025
Third round of review:	Number of reviewers: 1<br>Accepted : Dec 10, 2025
Data freely available:	NA
Code freely available:	NA

This transparent peer review record is not systematically proofread, type-set, or edited. Special characters, formatting, and equations may fail to render properly. Standard procedural text within the editor's letters has been deleted for the sake of brevity, but all official correspondence specific to the manuscript has been preserved.

Referees' reports, first round of review

Reviewer #1:

The authors set out to challenge the current paradigm that monogenic inborn errors of immunity (IEI) can only result from rare (<1%) pathogenic alleles. This MAF is indeed a commonly applied threshold when interrogating sequencing data for IEI-relevant pathogenic alleles in either clinical diagnostic or research contexts. The subject matter is therefore important and the review timely, given recent discoveries. It is also provocative and arguably misleading in certain areas, so I would urge the authors to consider the following points if revising their manuscript.

1. The majority of the review is given over to the discussion, one by one, of "common" alleles putatively linked to monogenic IEI, in chronological order of their discovery. In my view, a more helpful structure would instead group and organise these variants to highlight shared reasons underlying the apparently paradoxical association of common alleles with IEI that have been assumed to be rare. The abstract teases the reader with a numbered list of potential mechanisms - however these themes are not systematically developed within the review that follows.
2. The authors adopt an inclusive approach that embraces incompletely penetrant risk alleles for relatively prevalent conditions such as Crohn's disease (NOD2) or "autoimmunity" (PTCRA) alongside 100% penetrant pathogenic alleles for extreme and rare immunological phenotypes including SCID/CID (DCLRE1C, IKBKB). At the same time, GWAS is unreasonably dismissed, as for example in the final paragraph: "The limitation imposed by the need to correct for multiple testing and the lack of experimental support are intrinsic and inevitable limitations of the GWAS approach". I am missing a statistical genomic analysis that places these genetic risks of varying effect size within a unifying framework that respects the complementarity of alternative investigative methods.
3. Related to the above, there is almost no discussion of the importance of accurately ascertaining population prevalence of a given immunological disorder in relation to the maximum MAF expected for a fully penetrant pathogenic allele. That pathogenic alleles of MEFV or Artemis deficiency are common in those populations that also suffer much higher prevalence of the corresponding diseases is entirely expected.
4. When it comes to incomplete penetrance, the choice of a lower limit OR of 5 for a "monogenic" effect seems very low and just as arbitrary as the upper MAF threshold of 1% with which the authors take issue. The whole concept of penetrance is problematic when it comes to susceptibility to infectious disease, dependent as it must be on exposure to the relevant pathogen. Does it matter what proportion of sufferers from a particular "disease" bear pathogenic alleles of a designated gene and/or whether variants in that gene alone are sufficient to confer disease, independently of other risk factors? I think there is a broader discussion to be had here about what constitutes a monogenic disease and the utility of identifying genetic drivers, both for the individual/family and in terms of scientific understanding. Badging incompletely penetrant HLA-associated autoimmune diseases as "monogenic" does not seem helpful.

Reviewer #2:

Drs. Beziat and Casanova review the topic of Common genetic variants associated with clinically relevant functional effects. They compare these with the conventional concept that rare genetic variants are more likely to be the cause of clinical disease. They focus the review immune diseases, their area of expertise, but the concept is relevant to any group of genetic diseases where the pathogenicity of rare vs. common genetic variants is an area of ongoing controversy and study.

Overall, the review is thorough, well referenced, and generally well written. There are a couple of rough sections that don't flow as well as they could and revisions of the text are suggested below. I appreciate that for most of the disorders the review that are associated with common variants, they provide both a historical perspective of when the disease was described and when the gene association was made complete with citations of original papers. For most sections, the authors also speculate about potential events/conditions that could have driven selection for or against each common variant.

I'm well acquainted with the concept of penetrance but the description of penetrance in the 2nd and 3rd sentences of the introduction is perplexing and required multiple reads to understand what the authors were trying to convey. It's a rough way to start an otherwise excellent review and I'm concerned that readers may be discouraged from continuing on into the remainder of the review. I'd suggest that the authors revisit and try to clarify/simplify this section.

The section of the introduction on page 3 that gives 6 potential reasons for incomplete penetrance is an important conceptual section of the review and sets up the background for the detailed descriptions of each disorder that follows. This would lend itself to a figure that succinctly summarizes these 6 reasons/concepts. Please consider adding - I think it would enhance the paper.

In the 2nd paragraph of section 1 "Common variants at HLA loci underlie autoimmune conditions" the authors discuss the risks of AS in HLA-B27 positive individuals and suggest it may have been selected for due to a potential benefit in responses to infections. Since the question of infection responses is raised here, I'd suggest that the authors add a comment that in addition to the increased risk for AS, HLA-B27 positivity dramatically increases the risk of post-infectious reactive arthritis.

In section 3 "C6 and C9 deficiency", the 2nd paragraph begins "C9 deficiency of the paragraph that begins "C9 Deficiency was first described...." the second sentence reads "Indeed, during the formation of the CAM..." I think this was intended to be "the MAC" not the CAM - double-check

In the last paragraph of the paper's discussion section, the sentence "Auto-Abs against cytokines were already known to affect millions of people" seems out of place here and may be either deleted or moved."

Reviewer #3:

In the manuscript, Beziat and Casanova present a review of known monogenic disorders of immunity that are caused by common alleles. An important concept that is usually not considered when these group of disorders are studies. The paper is well researched, comprehensive, and clearly written. The message is important and timely. However, the introduction lacks clarity and the discussion can be expanded to help the different groups of researchers involved in the filed collaborate more closely across methodological and genetic architecture boundaries. Additionally, a number of references to previous work are missing. Please find my recommendations below.

Introduction: I find the introduction factually correct but unfocused and hard to follow. The authors do not get to the main point of the paper (high MAF variants that can cause IEIs) until page four! Even though they bring lots of examples that (if read by a - fully awake- genetics expert) can lead to their main points. I think the introduction can be revised and tightened. Some suggestions are:
- Even though I agree with the authors arguments around penetrance [Gaining an understanding of … SMAD6 alleles)] I think this section is loosely connected with the rest of the introduction. Is the idea to imply incomplete penetrance is common in monogenetic diseases? Or that is is one possible cause that pathogenic alleles can rise to high MAF? If the first, I think this section can be added in a box in the paper because it is not directly related to the question of MAF. If the later this should be stated clearly and also added to the abstract and the rest of the paper and one of the reasons underlying high-MAF pathogenic variants.
- Similarly, the section on the RR and OR, while correct it diverts from the main focus of the paper what is the point of this section and how does it relate to the main focus of the paper? What is the main conclusion form that section as it relates to the main point of the review? Is the example necessary in the text or the RR, OR example can be put into box within the paper?
- All in all, the message of pages 2 and 3 seems to be not all pathogenic variants are fully penetrant or have high effect sizes. If I try to add personal knowledge to this and read in between the lines, this rationally leads to: so not all pathogenic variants are under heavy negative selection nor all of them are pathogenic in all contexts and thus they can reach high MAFs. If this is the message the authors can state it sooner and clearer to avoid a diluted rational or hidden links that the reader must excavate.
- The fact that neither of these two points (penetrance and effect size) is part of the four reasons mentioned in the abstract adds to the confusion. Should they be added? How do these points relate to bottlenecks, drift, slow purging, and balancing selection when it comes to high MAF pathogenetic variants in IEI?

Individual examples:
- HLA: References 25-27 do not include more recent finding on the relationship between HLA and possible selection conferred by pathogens in ancient humans. Please add these references individually, one example include https://doi.org/10.1038/s41586-023-06618-z
- C2: Needs references: "Patient-based studies led to the discovery of common genetic defects of complement", "Genetic deficiencies of MAC components underlie invasive disease due to Neisseria.", "Complete deficiencies of alternative pathway proteins confer a predisposition to invasive bacterial infections"

- NOD2: "Population-based studies have shown Crohn's disease — a chronic inflammatory bowel disease (IBD) characterized by patchy intestinal inflammatory lesions in the gastrointestinal tract leading to chronic abdominal pain, diarrhea, obstruction and/or
- perianal lesions — to have a monogenic origin". Monogenic CD cases are a small % of total patient population. The sentence is misleading as is, please adjust to make clear most CD cases are in fact polygenic/complex. The paper the authors reference makes this clear and explicitly says in the abstract "Crohn's disease is a complex disease".
- SCID: "The high frequency of these alleles in Native American populations is unlikely to result from balancing selection. It probably results from genetic drift, with isolation or bottlenecks followed by rapid expansion of the corresponding populations." This is known as founder effect, please add to this section. (see: https://www.pnas.org/doi/full/10.1073/pnas.0903341106).
- Type I interferon: The examples of IEI impairing the type I IFN pathway lack references susceptibility to common respiratory viruses found before the ones in relation to covid-19. Please add the relevant references (https://pubmed.ncbi.nlm.nih.gov/28716935/ and https://pubmed.ncbi.nlm.nih.gov/28606988/).

- Missing example: G6PD deficiency and its relationship with diabetes in African and African American populations is another example that fits with the subject of this paper. Please add. (For more information see: https://pubmed.ncbi.nlm.nih.gov/38918629/).

Conclusion: I agree with the authors that non-additive models, particularly recessive models, should be considered in GWAS. However, I believe GWAS deserves a more expanded discussion. While GWAS has inherent limitations, some of which the authors mention, it remains a powerful tool for identifying causal variants, genes, and loci, when combined with appropriate functional follow up - particularly for the types of variants discussed in this paper. This is increasingly true in light of the rapid growth in GWAS sample sizes and diversity, driven by expanding biobank resources and decreasing data generation costs. Furthermore, modern GWAS no longer rely solely on minor allele frequency thresholds but instead use minor allele count cutoffs, which allow for well-calibrated test statistics and improved power, aligning with the authors' argument for avoiding arbitrary MAF cutoffs.
There are numerous examples of GWAS supporting genes or variants implicated in inborn errors of immunity (IEI), independent of IEI-focused studies. For instance, many HLA alleles have been identified through GWAS; TYK2 variants linked to autoimmunity were discovered before and outside of its known role in TB; and a recent study associated MVEF with ankylosing spondylitis in Middle Eastern populations (https://pubmed.ncbi.nlm.nih.gov/30946743/). I encourage the authors to explore their list of 14 genes/loci in the GWAS Catalog, they will find additional supporting examples.
Finally, some of the challenges related to conducting ancestry- or population-specific analyses, highlighted by the authors, have already been addressed within the GWAS and admixture mapping fields. Established approaches for accounting for genetic similarity and diversity in multi-ancestry cohorts can be adapted to patient-based studies as well.
All in all, expanding the discussion to include the complementarity of GWAS, patient/family-based studies, and experimental approaches would provide a more accurate representation of the field's evolution. It would also underscore how discovery rates can accelerate when researchers collaborate across methodological and genetic architecture boundaries. The authors are well positioned to highlight this important perspective.

Additional comments:
- Please number your pages and lines, it is really difficult to comment on a long manuscript without appropriate styling to help the reviewers.
- In the introduction the authors state "…although there is no need to explain healthy cases with the detrimental genotype if all sick relatives carry an at-risk genotype that can be mechanistically connected with immunological and clinical phenotypes." While I agree than healthy individuals can carry putative or established causal variants, I disagree with this view that there is no need to further investigate the underlying causes of the variability in phenotype. Trying to understand this phenomenon has extended our understanding of genome structure and function for example the effect of regulatory variants in limiting shaping the penetrance of pathogenic coding variants (see 10.1038/s41588-018-0192-y). Please revise this sentence.
- "A disease can be considered monogenic, even with low penetrance, provided that it is strongly associated with a monogenic genotype, with this association supported by experimental evidence." Please add appropriate references, one possible supporting study can be 10.1001/jama.2021.23686 which shows that most pathogenetic variants have indeed small ORs/effect sizes.
- "However, a handful of monogenic immunological conditions were discovered in large population-based studies. In these studies, focusing on common conditions, the involvement of a common allele was expected." Please add references.

Authors' response to the first round of review

Authors: We warmly thank the reviewers for their positive and constructive evaluation. We revised our manuscript taking their helpful and insightful comments into account.

Reviewer #1: The authors set out to challenge the current paradigm that monogenic inborn errors of immunity (IEI) can only result from rare (<1%) pathogenic alleles. This MAF is indeed a commonly applied threshold when interrogating sequencing data for IEI-relevant pathogenic alleles in either clinical diagnostic or research contexts. The subject matter is therefore important and the review timely, given recent discoveries.

Authors: We warmly thank the reviewer.

It is also provocative and arguably misleading in certain areas, so I would urge the authors to consider the following points if revising their manuscript.

1. The majority of the review is given over to the discussion, one by one, of "common" alleles putatively linked to monogenic IEI, in chronological order of their discovery. In my view, a more helpful structure would instead group and organise these variants to highlight shared reasons underlying the apparently paradoxical association of common alleles with IEI that have been assumed to be rare. The abstract teases the reader with a numbered list of potential mechanisms - however these themes are not systematically developed within the review that follows.

Authors: We thank Reviewer #1 for this thoughtful suggestion. We agree that grouping genotypes based on the evolutionary causes of their elevated frequencies—if these causes were known with certainty—would be valuable for readers. We had initially considered such a classification in an earlier version of our review. For example, the high frequency of at-risk alleles in IKBKG, DCLRE1C, ADA, IFNAR1, and IFNAR2 probably reflects historical bottlenecks followed by rapid population expansion (founder effects). In contrast, the elevated frequency of at-risk MEFV and CCR5 variants is probably due to past balancing selection, providing a selective advantage to heterozygous carriers against an as-yet uncertain pathogenic threat. However, for other at-risk genotypes (C2, C6, C9, NOD2, PTCRA, TYK2, TIM3), the evolutionary causes remain unclear; we thus prefer not to assign them to specific categories. Instead, we revised our paper by grouping them in two main categories based on disease penetrance in at-risk genotype carriers (see below). In addition, in the introduction, we now describe the possible mechanisms explaining the high frequency of the deleterious alleles.

2. The authors adopt an inclusive approach that embraces incompletely penetrant risk alleles for relatively prevalent conditions such as Crohn's disease (NOD2) or "autoimmunity" (PTCRA) alongside 100% penetrant pathogenic alleles for extreme and rare immunological phenotypes including SCID/CID (DCLRE1C, IKBKB). At the same time, GWAS is unreasonably dismissed, as for example in the final paragraph: "The limitation imposed by the need to correct for multiple testing and the lack of experimental support are intrinsic and inevitable limitations of the GWAS approach". I am missing a statistical genomic analysis that places these genetic risks of varying effect size within a unifying framework that respects the complementarity of alternative investigative methods.

Authors: We thank reviewer #1 for raising this important point. As stated in the introduction, there is no universally accepted “monogenic threshold”. While it is true that the variants in NOD2, PTCRA, TIM3, and CCR5 included in our review are far from being completely penetrant for the related immunological phenotypes, they confer odd ratios (OR) above our arbitrary threshold (>5), and causality is further supported by experimental evidence. As pointed by reviewer #1 our threshold is inclusive: we do not restrict our review to Mendelian disorders (i.e. monogenic with full penetrance). To our knowledge, beyond NOD2 and HLA alleles, no alleles identified in GWAS studies or populationbased studies of immune-related phenotypes reach our OR threshold of 5. Would the reviewers know relevant examples. we would be glad to include them. In addition, we toned down the sentence quoted by the reviewer, and reorganized our manuscript, with variants displaying high penetrance and risk vs. variants displaying low penetrance but high risk.

3. Related to the above, there is almost no discussion of the importance of accurately ascertaining population prevalence of a given immunological disorder in relation to the maximum MAF expected for a fully penetrant pathogenic allele. That pathogenic alleles of MEFV or Artemis deficiency are common in those populations that also suffer much higher prevalence of the corresponding diseases is entirely expected.

Authors: Thank you for this important suggestion. We now stress this important notion in the revised discussion of our manuscript.

4. When it comes to incomplete penetrance, the choice of a lower limit OR of 5 for a "monogenic" effect seems very low and just as arbitrary as the upper MAF threshold of 1% with which the authors take issue. The whole concept of penetrance is problematic when it comes to susceptibility to infectious disease, dependent as it must be on exposure to the relevant pathogen. Does it matter what proportion of sufferers from a particular "disease" bear pathogenic alleles of a designated gene and/or whether variants in that gene alone are sufficient to confer disease, independently of other risk factors? I think there is a broader discussion to be had here about what constitutes a monogenic disease and the utility of identifying genetic drivers, both for the individual/family and in terms of scientific understanding. Badging incompletely penetrant HLA-associated autoimmune diseases as "monogenic" does not seem helpful.

Authors: The definition of a monogenic effect is indeed important and challenging. As discussed in our revised introduction, we define monogenic inheritance based on a strong association (OR >5), which encompasses HLAassociated autoimmune diseases. This is admittedly inclusive. It does not exclude epistasis as a possible hypothesis for explaining low penetrance of some risk alleles. The possible mechanisms– in other words the “other risk factors”–are now discussed in Box 2, together with the mechanisms explaining incomplete penetrance.

Reviewer #2: Drs. Beziat and Casanova review the topic of Common genetic variants associated with clinically relevant functional effects. They compare these with the conventional concept that rare genetic variants are more likely to be the cause of clinical disease. They focus the review immune diseases, their area of expertise, but the concept is relevant to any group of genetic diseases where the pathogenicity of rare vs. common genetic variants is an area of ongoing controversy and study.

Overall, the review is thorough, well referenced, and generally well written. There are a couple of rough sections that don't flow as well as they could and revisions of the text are suggested below. I appreciate that for most of the disorders the review that are associated with common variants, they provide both a historical perspective of when the disease was described and when the gene association was made complete with citations of original papers. For most sections, the authors also speculate about potential events/conditions that could have driven selection for or against each common variant.

Authors: We wamly thanks the referee.

1. I’m well acquainted with the concept of penetrance but the description of penetrance in the 2nd and 3rd sentences of the introduction is perplexing and required multiple reads to understand what the authors were trying to convey. It’s a rough way to start an otherwise excellent review and I’m concerned that readers may be discouraged from continuing on into the remainder of the review. I’d suggest that the authors revisit and try to clarify/simplify this section.

Authors: We thank reviewer #2 for this suggestion. Indeed, the previous version of our introduction was too complex. We have revised our introduction extensively, including the first sentences on penetrance. See below for more details.

2. The section of the introduction on page 3 that gives 6 potential reasons for incomplete penetrance is an important conceptual section of the review and sets up the background for the detailed descriptions of each disorder that follows. This would lend itself to a figure that succinctly summarizes these 6 reasons/concepts. Please consider adding - I think it would enhance the paper.

Authors: Thank you for this suggestion. Creating a figure summarizing these 6 different concepts is difficult, as each of them would deserve an independent figure. Instead we created a box summarizing these concepts (Box 2), as suggested by reviewer #3. This modification is in line with the first comment of reviewer #2. We think it improves our introduction.

3. In the 2nd paragraph of section 1 "Common variants at HLA loci underlie autoimmune conditions" the authors discuss the risks of AS in HLA-B27 positive individuals and suggest it may have been selected for due to a potential benefit in responses to infections. Since the question of infection responses is raised here, I'd suggest that the authors add a comment that in addition to the increased risk for AS, HLA-B27 positivity dramatically increases the risk of post-infectious reactive arthritis.

Authors: We thank reviewer #2 for this great suggestion. We now include a comment on the increased risk of reactive arthritis in HLA-B27 positive carriers.

4. In section 3 "C6 and C9 deficiency", the 2nd paragraph begins "C9 deficiency of the paragraph that begins "C9 Deficiency was first described...." the second sentence reads "Indeed, during the formation of the CAM..." I think this was intended to be "the MAC" not the CAM - double-check.

Authors: We thank the reviewer for pointing this typo. It is now corrected.

5. In the last paragraph of the paper's discussion section, the sentence "Auto-Abs against cytokines were already known to affect millions of people" seems out of place here and may be either deleted or moved." **Authors**: We agree with reviewer #2, the sentence was deleted.

Reviewer #3: In the manuscript, Beziat and Casanova present a review of known monogenic disorders of immunity that are caused by common alleles. An important concept that is usually not considered when these group of disorders are studies. The paper is well researched, comprehensive, and clearly written. The message is important and timely.

Authors: We warmly thank the reviewer.

However, the introduction lacks clarity and the discussion can be expanded to help the different groups of researchers involved in the filed collaborate more closely across methodological and genetic architecture boundaries. Additionally, a number of references to previous work are missing. Please find my recommendations below.

1. Introduction: I find the introduction factually correct but unfocused and hard to follow. The authors do not get to the main point of the paper (high MAF variants that can cause IEIs) until

page four! Even though they bring lots of examples that (if read by a - fully awake- genetics expert) can lead to their main points. I think the introduction can be revised and tightened.

Authors: We thank the referee for this very helpful comment, which is in agreement with comment 1 of reviewer #2. We extensively revised and trimmed the introduction, while adding two Boxes.

Some suggestions are:
2. Even though I agree with the authors arguments around penetrance [Gaining an understanding of … SMAD6 alleles)] I think this section is loosely connected with the rest of the introduction. Is the idea to imply incomplete penetrance is common in monogenetic diseases? Or that is is one possible cause that pathogenic alleles can rise to high MAF? If the first, I think this section can be added in a box in the paper because it is not directly related to the question of MAF. If the later this should be stated clearly and also added to the abstract and the rest of the paper and one of the reasons underlying high-MAF pathogenic variants.

Authors: We thank reviewer #3 for this great suggestion. We now include the possible causes of incomplete penetrance in Box 2.

3. Similarly, the section on the RR and OR, while correct it diverts from the main focus of the paper what is the point of this section and how does it relate to the main focus of the paper? What is the main conclusion form that section as it relates to the main point of the review? Is the example necessary in the text or the RR, OR example can be put into box within the paper?

Authors: We thank reviewer #3 for yet another great suggestion. We now include the section on size effect, OR and RR, as well as the relationship between RR and penetrance, in Box 1.

4. All in all, the message of pages 2 and 3 seems to be not all pathogenic variants are fully penetrant or have high effect sizes. If I try to add personal knowledge to this and read in between the lines, this rationally leads to: so not all pathogenic variants are under heavy negative selection nor all of them are pathogenic in all contexts and thus they can reach high MAFs. If this is the message the authors can state it sooner and clearer to avoid a diluted rational or hidden links that the reader must excavate.

Authors: We thank reviewer #3 for this suggestion. We now clearly state in the revised introduction why evolutionarily the alleles of interest can be at high MAFs in a given population.

5. The fact that neither of these two points (penetrance and effect size) is part of the four reasons mentioned in the abstract adds to the confusion. Should they be added? How do these points relate to bottlenecks, drift, slow purging, and balancing selection when it comes to high MAF pathogenetic variants in IEI?

Authors: We thank reviewer #3 for this suggestion. We now include the penetrance in the sentence relative to the slow purging.

Individual examples:

6. HLA: References 25-27 do not include more recent finding on the relationship between HLA and possible selection conferred by pathogens in ancient humans. Please add these references individually, one example include https://doi.org/10.1038/s41586-023-06618-z

Authors: We thank the reviewer for pointing us to this interesting research topic. We now quote a few example of positive selection at the HLA locus, including the suggested reference.

7. C2: Needs references: "Patient-based studies led to the discovery of common genetic defects of complement", "Genetic deficiencies of MAC components underlie invasive disease due to Neisseria.", "Complete deficiencies of alternative pathway proteins confer a predisposition to invasive bacterial infections".

Authors: We now include references for these three statements.

8. NOD2: "Population-based studies have shown Crohn's disease — a chronic inflammatory bowel disease (IBD) characterized by patchy intestinal inflammatory lesions in the gastrointestinal tract leading to chronic abdominal pain, diarrhea, obstruction and/or perianal lesions — to have a monogenic origin". Monogenic CD cases are a small % of total patient population. The sentence is misleading as is, please adjust to make clear most CD cases are in fact polygenic/complex. The paper the authors reference makes this clear and explicitly says in the abstract "Crohn's disease is a complex disease".

Authors: It is true that highly penetrant variants underlying inflammatory bowel diseases are rare, and were identified by patient based studies (e.g. IL10RB). Other variants, discovered by GWAS, have weak penetrance, sometime with an high OR, like NOD2. To our knowledge, the reason underlying the weak penetrance of NOD2 remains unknown. Genetic heterogeneity does not rule out the possible monogenic origin of a disease. It could be due to epistasis (digenic or polygenic), but experimental evidence is lacking. Disease penetrance in these patients may only require environmental factors, in line with an increasing disease prevalence worldwide.

Nevertheless, to clarify this point, we decided to split our review in two main sections:
- the pathogenic alleles with high penetrance
- the pathogenic alleles with high risk but low penetrance.

In addition, we tone down the quoted sentence to “Population-based studies have shown Crohn’s disease — a chronic inflammatory bowel disease (IBD) characterized by patchy intestinal inflammatory lesions in the gastrointestinal tract leading to chronic abdominal pain, diarrhea, obstruction and/or perianal lesions — can have a monogenic origin.”

This being said, we respectfully disagree with the referee’s statement that “most CD cases are in fact polygenic/complex”. These terms come from the field of population genetics and do not easily apply to clinical genetics. Whether any patient suffers from polygenic CD remains hypothetical: it has never been proven. As per complex CD, the lack of unambiguous definition of ‘complex’ prevents it to be tested.

9. SCID: "The high frequency of these alleles in Native American populations is unlikely to result from balancing selection. It probably results from genetic drift, with isolation or bottlenecks followed by rapid expansion of the corresponding populations." This is known as founder effect, please add to this section. (see: https://www.pnas.org/doi/full/10.1073/pnas.0903341106).

Authors: We thank the referee for this very helpful addition. We have modified the sentence accordingly as “The high frequency of these alleles in these populations is unlikely to result from balancing selection. It probably results from a founder effect, in other words a genetic drift, with isolation or bottlenecks followed by rapid expansion of the corresponding populations”.

10. Type I interferon: The examples of IEI impairing the type I IFN pathway lack references susceptibility to common respiratory viruses found before the ones in relation to covid-19. Please add the relevant references (https://pubmed.ncbi.nlm.nih.gov/28716935/ and https://pubmed.ncbi.nlm.nih.gov/28606988/).

Authors: We thank the referee for pointing this omission. We include these two references in our revised manuscript.

11. Missing example: G6PD deficiency and its relationship with diabetes in African and African American populations is another example that fits with the subject of this paper. Please add. (For more information see: https://pubmed.ncbi.nlm.nih.gov/38918629/).

Authors: We again thank the reviewer. This is an interesting example of yet another frequent disease-causing variant.

Variants underlying Glucose-6-phosphate dehydrogenase (G6PD) deficiency are divided in 5 main classes: Class 1--severe enzyme deficiency associated with chronic nonspherocytic hemolytic anemia.
Class 2--severe enzyme deficiency (less than 10%) associated with acute hemolytic anemia.
Class 3--moderate to mild enzyme deficiency (10-60%).
Class 4--very mild or no enzyme deficiency (60%).
Class 5—increased enzyme activity.

Only G6PD class 1 deficiency is considered as an IEI, and is included in the IUIS classification– within the congenital defects of phagocyte number or function.

The frequent variant in African and African American populations– known as A$^-$ (Val68Met + Asn126Asp)–belongs to Class 3 G6PD deficiency. This is the reason why we did not integrate G6PD variants in our review.

12. Conclusion: I agree with the authors that non-additive models, particularly recessive models, should be considered in GWAS. However, I believe GWAS deserves a more expanded discussion. While GWAS has inherent limitations, some of which the authors mention, it remains a powerful tool for identifying causal variants, genes, and loci, when combined with appropriate functional follow up - particularly for the types of variants discussed in this paper. This is increasingly true in light of the rapid growth in GWAS sample sizes and diversity, driven by

expanding biobank resources and decreasing data generation costs. Furthermore, modern GWAS no longer rely solely on minor allele frequency thresholds but instead use minor allele count cutoffs, which allow for well-calibrated test statistics and improved power, aligning with the authors' argument for avoiding arbitrary MAF cutoffs.

Authors: We understand the referee's point. While we doubt that population-based approaches can do better than family-based approaches to discover human genotypes underlying severe phenotypes, we do not know the future and we understand and respect the referee's hypotheses and hopes. Nevertheless, due to space constraints, and because the review is focused on monogenic disorders, we opted to not expand the discussion of GWAS. Yet, we revised our text to sound more agnostic.

13. There are numerous examples of GWAS supporting genes or variants implicated in inborn errors of immunity (IEI), independent of IEI-focused studies. For instance, many HLA alleles have been identified through GWAS; TYK2 variants linked to autoimmunity were discovered before and outside of its known role in TB; and a recent study associated MVEF with ankylosing spondylitis in Middle Eastern populations (https://pubmed.ncbi.nlm.nih.gov/30946743/). I encourage the authors to explore their list of 14 genes/loci in the GWAS Catalog, they will find additional supporting examples.

Authors: We thank the referee for this very helpful suggestion. We mined the GWAS catalog for 15 loci, as we added ADA deficiency in Somalia in our revised manuscript. A few genotype/phenotype associations had an OR above 5. However, these connections were not with immune phenotypes, and were therefore not included in our review (ADA and asceptic loosening; HAVCR2 and Oligodendroglioma; IFNAR1 and glioblastoma; IFNAR2 and Familial squamous cell lung carcinoma; IFNAR2 and Type 2 diabetes). As pointed by reviewer #3, we also found an association between the rs61752717 in MEFV and ankylosing spondylitis. Although this association has an OR of 4.8, we now quote it in our review.

14. Finally, some of the challenges related to conducting ancestry- or population-specific analyses, highlighted by the authors, have already been addressed within the GWAS and admixture mapping fields. Established approaches for accounting for genetic similarity and diversity in multi-ancestry cohorts can be adapted to patient-based studies as well.

Authors: We thank the referee for raising this point. We agree that adjustement to ancestry is helpful for various types of genetic association studies.

15. All in all, expanding the discussion to include the complementarity of GWAS, patient/family-based studies, and experimental approaches would provide a more accurate representation of the field's evolution. It would also underscore how discovery rates can accelerate when researchers collaborate across methodological and genetic architecture boundaries. The authors are well positioned to highlight this important perspective.

Authors: We thank the referee for this important point. We think that the referee's point is perfectly valid, yet goes probably beyond the scope of this review, which is focused on common variants underying monogenic IEI. In the future, we hope to write another review, ideally with a

population geneticist, which might help bridge the gap between patient- and population-based approaches.

Additional comments:
16. Please number your pages and lines, it is really difficult to comment on a long manuscript without appropriate styling to help the reviewers.

Authors: We thank the referee for this suggestion. We now include the page and line numbers.

17. In the introduction the authors state "…although there is no need to explain healthy cases with the detrimental genotype if all sick relatives carry an at-risk genotype that can be mechanistically connected with immunological and clinical phenotypes." While I agree than healthy individuals can carry putative or established causal variants, I disagree with this view that there is no need to further investigate the underlying causes of the variability in phenotype. Trying to understand this phenomenon has extended our understanding of genome structure and function for example the effect of regulatory variants in limiting shaping the penetrance of pathogenic coding variants (see 10.1038/s41588-018-0192-y). Please revise this sentence.

Authors: We apologize for the misunderstanding, probably accounting for the complexity of our previous introduction, as pointed by all three reviewers. We agree with the reviewer regarding the importance of investigating the healthy carriers of at risk genotypes. We wrote in the previous and current version of the paper: "Gaining an understanding of the mechanisms underlying the incomplete penetrance of monogenic disorders is a major endeavor in the field of IEI." We also want to thank the reviewer for pointing us to this interesting study. Although this 2018 paper adds weight to the 1997 hypothesis (McGee et al, AJHG; doi: 10.1086/301614), The proposed mechanism is not directly supported by evidence within a well-characterized disease context.. We nevertheless cite these two papers among the proposed causes of incomplete penetrance.

18. "A disease can be considered monogenic, even with low penetrance, provided that it is strongly associated with a monogenic genotype, with this association supported by experimental evidence." Please add appropriate references, one possible supporting study can be 10.1001/jama.2021.23686 which shows that most pathogenetic variants have indeed small ORs/effect sizes.

Authors: We thank the reviewer for pointing us to this interesting manuscript. We are not convinced by the authors' claims, as their study encompasses a very broad range of both loci and alleles. While some genes and alleles have been mechanistically and causally characterized in great depth, most genes and alleles have not. It is therefore difficult to claim that most pathogenic variants have low penetrance. It is more accurate to say that most alleles claimed to be pathogenic, with insufficient evidence, do not display high penetrance and even that some or perhaps many might not be pathogenic at all. We nevertheless cite this paper in the corresponding section.

19. "However, a handful of monogenic immunological conditions were discovered in large population-based studies. In these studies, focusing on common conditions, the involvement of a common allele was expected." Please add references.

Authors: We thank the referee and now include references.

Referees' reports, second round of review

Reviewer #3:

Thank you for addressing the concerns raised. One area I would urge you to revise more strongly is the treatment of GWAS. While your caution about its limitations is valid, the current tone can come across as dismissive. Given the central role GWAS plays in human genetics, a more balanced framing — one that acknowledges its complementarity with family-based and mechanistic studies, would make the manuscript both more accurate and more broadly useful to readers across disciplines.

Authors' response to the second round of review

Authors: We thank again the reviewers for their positive and constructive evaluation during the revision process.

Reviewer #3: Thank you for addressing the concerns raised. One area I would urge you to revise more strongly is the treatment of GWAS. While your caution about its limitations is valid, the current tone can come across as dismissive. Given the central role GWAS plays in human genetics, a more balanced framing — one that acknowledges its complementarity with family-based and mechanistic studies, would make the manuscript both more accurate and more broadly useful to readers across disciplines.

Authors: We do not mean do be dismissive about GWAS studies. After carefully reading the manuscript we have modified two sentences.

1. Page 4, line 63:
"Most monogenic disorders of immunity were discovered via patient- and family-based studies or studies of rare conditions in the field of IEI. **However, a handful of monogenic immunological conditions were discovered in large population-based studies**." This sentence was replaced by:
"Most monogenic disorders of immunity were discovered via patient- and family-based studies or studies of rare conditions in the field of IEI. **However, a subset of monogenic immunological conditions were discovered in large population-based studies**"

2. Page 27, line 576:

“Excluding HLA, which merits its own separate analysis, we have identified 17 common variants at 13 loci. This number is relatively small next to the >450 monogenic IEI due to rare variants discovered in patient-based studies. **However, it is a relatively large number next to the number of monogenic conditions discovered in population-based studies, as all such attempts led to the discovery of a common allele.”** This sentence was replaced by: “Excluding HLA, which merits its own separate analysis, we have identified 17 common variants at 13 loci. This number is relatively small next to the >450 monogenic IEI due to rare variants discovered in patient-based studies. **This probably results from the common practice of filtering out common alleles when searching for new IEI – and monogenic inborn errors at large.”**

Authors’ response to the third round of review

We have now added an additional sentence in the discussion outlining how population-based GWAS approaches and family-based studies can be integrated to identify and validate novel genotype–phenotype associations:

“*Recessive traits should be considered in genome-wide association studies of common variants (GWAS); they may be highly penetrant in a subset of, or the entire population sample. Candidate monogenic genotypes may then be investigated experimentally, through targeted functional and familial genetic studies.*”
